# Do Patients Want to Die at Home? A Systematic Review of the UK Literature, Focused on Missing Preferences for Place of Death

**DOI:** 10.1371/journal.pone.0142723

**Published:** 2015-11-10

**Authors:** Sarah Hoare, Zoë Slote Morris, Michael P Kelly, Isla Kuhn, Stephen Barclay

**Affiliations:** 1 Primary Care Unit, Department of Public Health and Primary Care, Cambridge Institute of Public Health, University of Cambridge School of Clinical Medicine, Forvie Site, Cambridge Biomedical Campus, Cambridge, United Kingdom; 2 University of Cambridge Medical Library, School of Clinical Medicine, Box 111, Cambridge Biomedical Campus, Cambridge, United Kingdom; University of Glasgow, UNITED KINGDOM

## Abstract

**Background:**

End-of-life care policy has a focus on enabling patients to die in their preferred place; this is believed for most to be home. This review assesses patient preferences for place of death examining: the extent of unreported preferences, the importance of patient factors (place of care and health diagnosis) and who reports preferences.

**Methods and Findings:**

Systematic literature review of 7 electronic databases, grey literature, backwards citations from included studies and Palliative Medicine hand search. Included studies published between 2000–2015, reporting original, quantifiable results of adult UK preferences for place of death. Of 10826 articles reviewed, 61 met the inclusion criteria. Summary charts present preferences for place of death by health diagnosis, where patients were asked and who reported the preference. These charts are recalculated to include ‘missing data,’ the views of those whose preferences were not asked, expressed or reported or absent in studies. Missing data were common. Across all health conditions when missing data were excluded the majority preference was for home: when missing data were included, it was not known what proportion of patients with cancer, non-cancer or multiple conditions preferred home. Patients, family proxies and public all expressed a majority preference for home when missing data were excluded: when included, it was not known what proportion of patients or family proxies preferred home. Where patients wished to die was related to where they were asked their preference. Missing data calculations are limited to ‘reported’ data.

**Conclusions:**

It is unknown what proportion of patients prefers to die at home or elsewhere. Reported preferences for place of death often exclude the views of those with no preference or not asked: when ‘missing data’ are included, they supress the proportion of preferences for all locations. Caution should be exercised if asserting that most patients prefer to die at home.

## Introduction

English health policy has as one of its aims the goal of enabling patients to die in their preferred place, which is believed for most to be their home [1], although whether home is always the best and preferred place of death is of increasing debate [2]. Healthcare professionals are encouraged to record end of life care (EOLC) patients’ preferred place of death (PPOD) [3], with percentage of home deaths and deaths in preferred place regarded as a key performance indicator of EOLC services [4].

Home is stated to be where most patients want to die because, as the foreword of the 2008 EOLC Strategy reports, “From surveys of the *general public* we know that, given the opportunity and right support, most people would prefer to die at home.[1]” The extent to which the preferences of the general public reflect the views of *dying patients* is unclear; the two viewpoints may vary due to differences in priorities [5] and whether preferences asked are hypothetical or of practical significance [6].

It is also unknown how ‘missing’ preferences (the views of participants with no clear preference, or who were unwilling or unable to express or communicate a preference) are treated [7]. Patient preferences for place of death are often ill-defined and evolve as their health deteriorates and their needs change [8]. Preferences for place of death are not categorical choices; they are highly contingent and dependent on the support available [9]. Respondents to questionnaires about preferences are typically though restricted to choices of home, hospice, care home or hospital, whether the questions are asked of the general public [10] or dying patients [11] and it is unclear how those whose preferences do not fit these choices are included in study reporting. Excluding ‘missing’ preferences, while not a problem of the magnitude as that of unpublished clinical trials [12], has the potential to significantly misrepresent patient views and hide the nuances of the PPOD decision-making process.

We therefore undertook a new systematic review of literature concerning the preferences of UK respondents for place of death, with a particular focus on the inclusion of “missing preferences”- participants excluded from analysis since their preference was not asked, expressed or reported. Higginson and Sen-Gupta’s [13] 2000 international review of PPOD of advanced cancer patients is widely cited in the early EOLC literature and reflects the attention at that time on cancer care. Unlike that review we included all diagnoses to follow the current focus on palliative care for all patients, and restricted the literature to UK populations in order to standardise the context. We explored the variation in preferences by participant’s health condition, who reported the preference and where they were asked their preferences. Since others have reported few high quality UK papers on preferences for place of death since 2000 [6], we applied a well-respected study quality measure and included the ‘grey literature’[14].

## Methods

Preferences were examined in three ways; whether the participant had a malignant diagnosis, whose preference was reported, and where participants were asked their preference. The role of disease is pertinent because UK EOLC provision has historically focused on meeting the needs of cancer patients [1], and therefore it is plausible that patients with other conditions may have different end of life preferences which are not as well recognised. The role of a participant could have an implication on the answer given when asked about PPOD: how preferences are considered may be different for a dying patient compared to a family member acting as a proxy for a dying or deceased patient or a member of the public [15]. The place of an EOLC participant’s care could also be related to their PPOD; patients experiences of care settings have been shown to be a contextualising factor in where they choose to die [9]. Since place of care is not always documented, we used the proxy measure of where participants were asked their preference.

The review questions were therefore:

- Are there differences in the preferences for dying at home in cancer patients compared to patients with other conditions?
- Are there differences in the preferences for dying at home as reported by patients, family members, health care professionals or the general public?
- Are there differences in the preferences for dying at home by where participants are asked their preference?
- What is the extent of missing data on these reported preferences?

### Search terms

**Fig 1:**
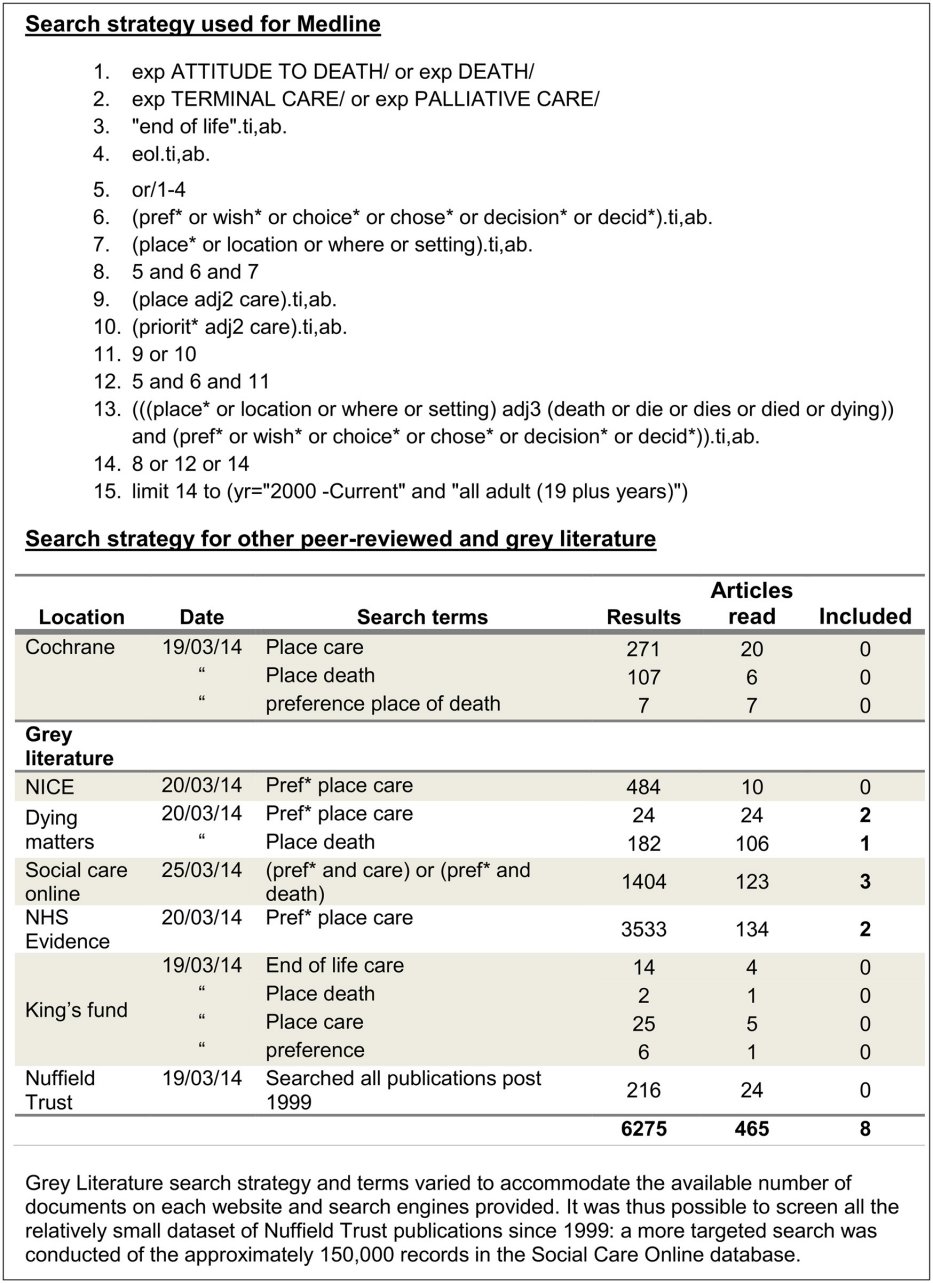
Search Strategy.

An initial scoping search was carried out (see S1 Fig for initial search strategy) and the results were reviewed with the below inclusion/exclusion criteria. The search strategy was then revised and improved with the guidance of the review team’s Information Scientist (IK) (see Fig 1 for search strategy). Searches for papers published between 2000 and January 2015 were carried out in Medline, Embase, PsycINFO (all via OVID), CINAHL (via EbscoHOST), Web of Science, Scopus, ASSIA (via Proquest) and the results were reviewed with the same criteria. A comprehensive search of grey literature identified other studies published over this period; relevant databases and websites of government policy, policy institutes and charities were reviewed.

Hand search of *Palliative Medicine*, the most prevalent journal for included articles, screening of relevant review papers, citation searches of all included papers for other peer reviewed papers, and the authors’ prior knowledge completed the search strategy.

### Inclusion and exclusion criteria

Articles were included in the review if they were: published after 1999, written in English, conducted in a UK setting, and reported quantifiable, empirical data on adults’ preferences for place of death. We restricted the literature to UK populations to increase the homogeneity of health service and cultural context. Likewise, we focused on adult preferences only, recognising the differences in EOLC for children [1]. Opinion pieces, conference abstracts and news reports were excluded unless they contained original empirical data.

### Selection and evaluation of studies

**Fig 2:**
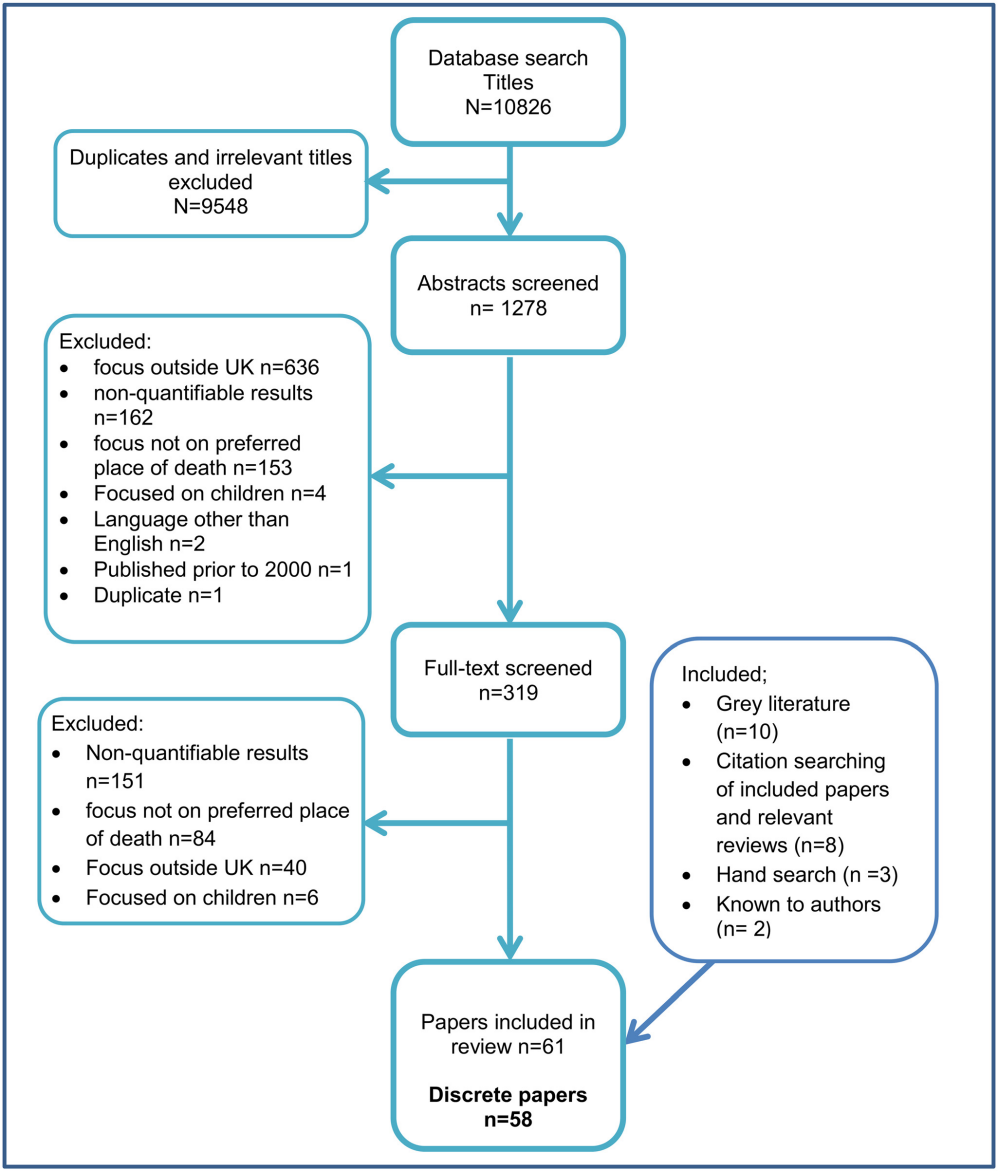
Flowchart of literature search.

The search results were downloaded into EndNote and duplicates removed. Titles were screened to remove irrelevant papers (by SH) and the included abstracts then reviewed by two authors independently (SH and ZM or SB), and the full text of potential papers obtained. Disagreements were resolved by discussion within the team. Fig 2 provides a flowchart of the literature search.

**Fig 3:**
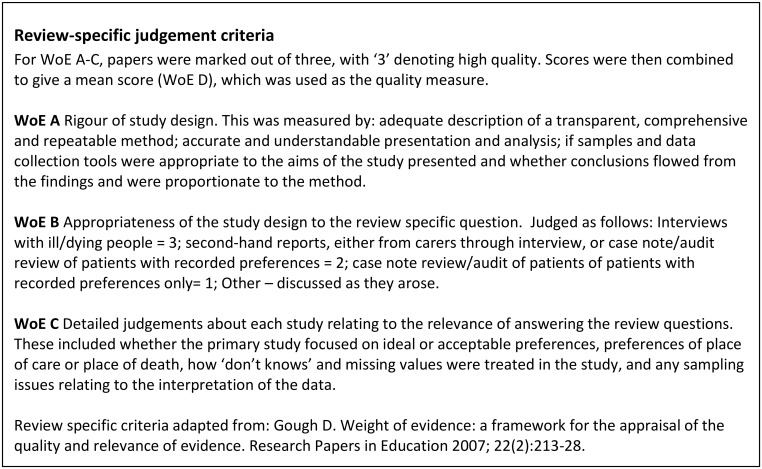
Gough’s Weight of Evidence Framework.

Each included paper was then weighted for its contribution towards answering the review question using Gough’s Weight of Evidence Framework [14]. Details of the Framework and the weighting method are shown in Fig 3. Each paper was weighted independently by two authors (SH and ZM or SB) with differences in scores reconciled through discussion. A sensitivity analysis of included papers investigated the impact of removing the lowest weighted papers but found no meaningful change in results; we therefore report analysis of all included papers.

### Data Synthesis

**Fig 4:**
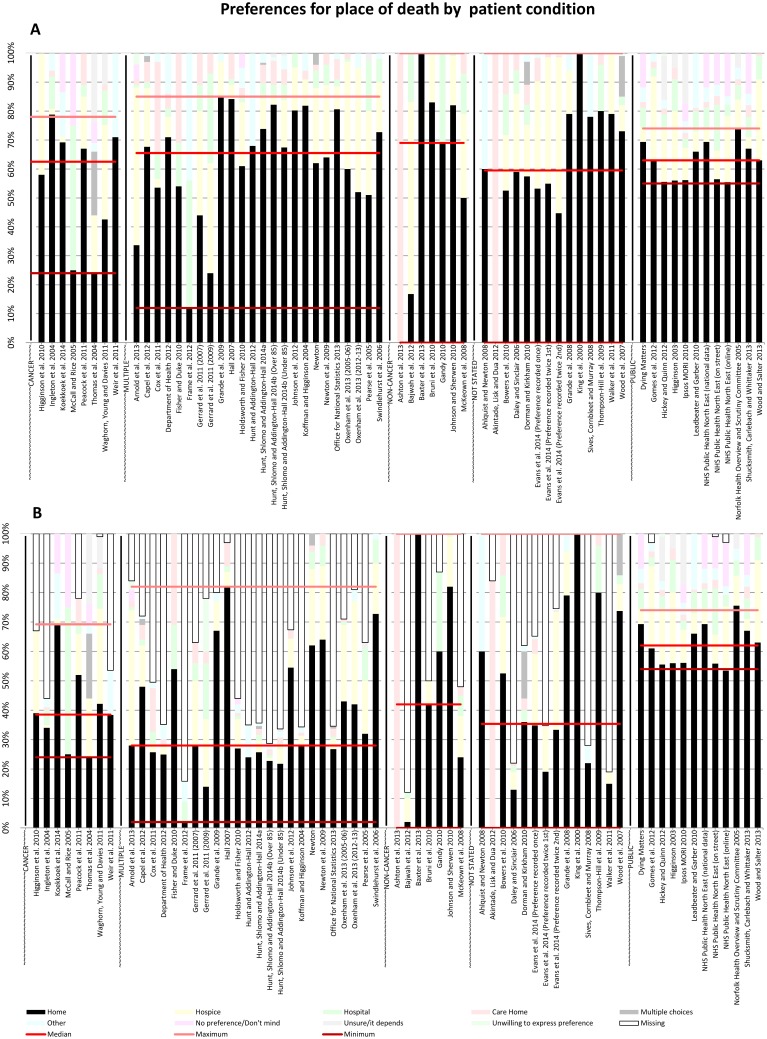
PPOD by patient condition (A) and PPOD by patient condition including missing data (B).

**Fig 5:**
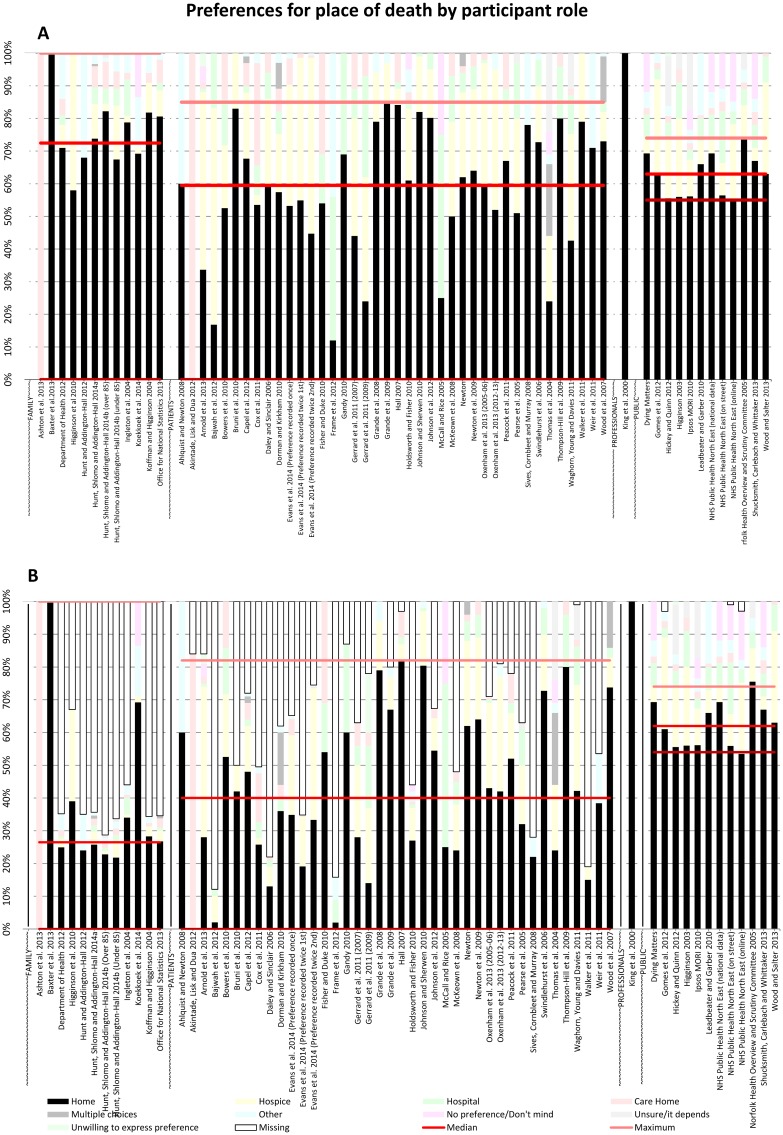
PPOD by participant role (A) and PPOD by participant role including missing data (B).

**Fig 6:**
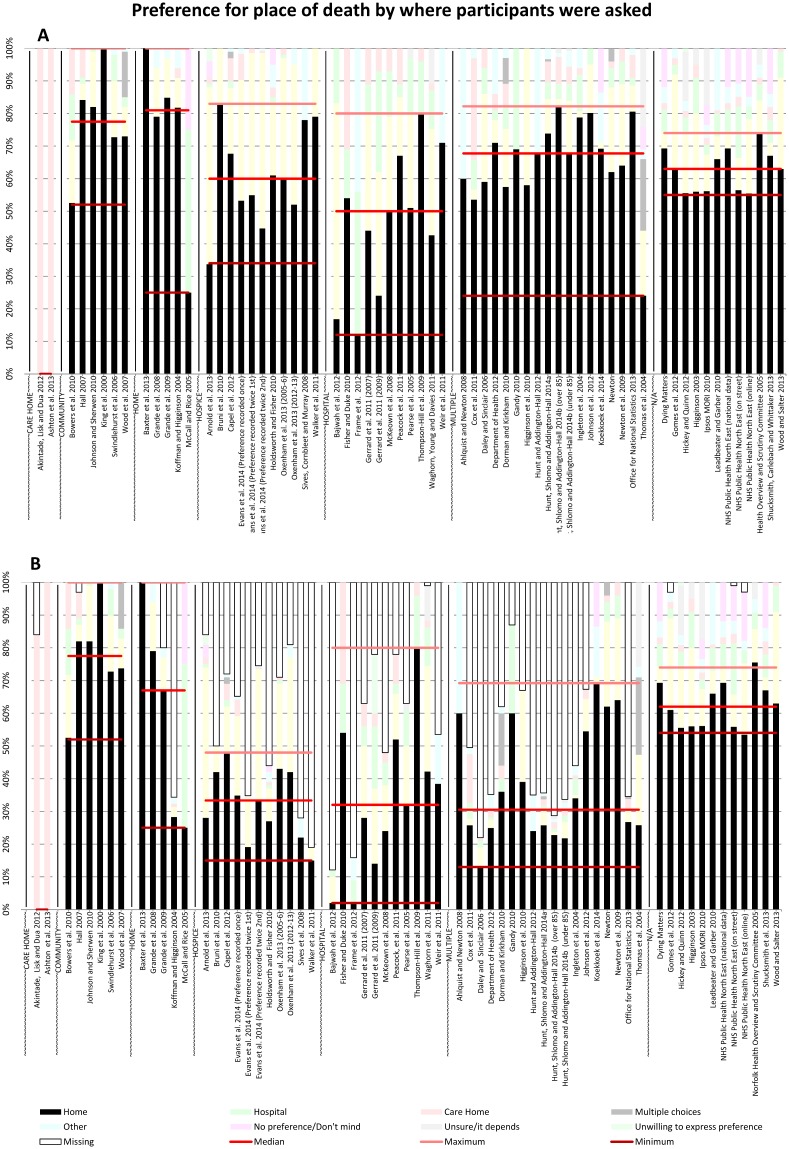
PPOD by where participants were asked (A) and including missing data (B).

Data were extracted from each included paper and tabulated in Microsoft Excel (by SH or ZM). Separate spreadsheets categorised studies by: main diagnosis of participants (cancer, non-cancer, multiple conditions, not stated and ‘public’ to refer to participants surveyed as a member of the general public); who reported the preference (patients, family or other informal carer, healthcare professionals and public) and setting (care home, home, hospice, hospital, multiple settings or ‘not applicable’ as participants were not patients or reporting on behalf of patients). To explore the impact of ‘missing data,’ these spreadsheets were then reproduced with preference percentages including participants whose preferences were either not recorded in the study or not reported in the paper. The ‘missing’ preferences were included as a discrete category since they could not accurately be included in any of the other pre-existing preference categories. Both sets of spreadsheets were plotted as bar charts with lines superimposed indicating median, maximum and minimum home preferences for each category (see Figs 4–6).

Where several different samples were reported in the papers, only that most relevant to the review were included. Where possible this was the ‘total’ preference for place of death of the entire sample; where this was not reported it was calculated where feasible from the available data. Where this was neither possible nor appropriate, for example where data were reported from different years, we report both populations. Preferences for ‘don’t mind’ and ‘no preference’ were merged. Likewise, where participants were not decided or were reported to have a preference of ‘changed mind,’ this was categorised as ‘Unsure/it depends.’ Reported non-responses were categorised as ‘missing’ unless a reason was given which meant that the response could be otherwise categorised (e.g. reported as ‘undecided’). References to nursing or residential home were categorised as ‘care home.’

Broad inclusion criteria ensured that all relevant data could be included and the review studies were diverse in terms of: populations and settings; sampling methods and sample sizes; and research methods. To test the data implications of this we stratified the sample by data source and found no evidence of systematic confounding (see S2 Fig). Due to the considerable heterogeneity it was not possible to undertake a meta-analysis to provide an overall percentage for home death preference, nor to test statistically the relationships between categories and preferences for place of death. As the data were not normally distributed, medians rather than means are presented.

## Results

### Overview of the study data

The initial search strategy yielded 1,973 titles, the full strategy 8,853 titles and the results were combined. The results of the grey literature search and additional searches were then incorporated at the end of reviewing process. 61 reports met the review inclusion criteria. Three reported duplicate data ([16, 17]; [18, 19]; and [20, 21]) and were combined for analysis leaving 58 discrete papers. Several studies reported datasets of multiple populations; three contained two datasets and therefore were included twice[22–24] and two studies generated three reports[25],[26] and were thus represented three times each. This brought the total number of included reports to 65.

Five reports were weighted as “high” using Gough’s ‘Weight of Evidence’ Framework [5, 7, 9, 27, 28]; 38 reports were weighted as “medium”, often due to their focus on the general public rather than participants close to the end the life. The remaining 15 reports were given a “low” weight; conference poster abstracts, letters to journals, grey literature reports of projects not designed to be of high academic standard, and studies where, for example, samples were not described or participant preference data were limited to home preferences alone. S1 Table presents summaries of the included reports.

The fifty-eight included studies were research papers (n = 34), poster abstracts (n = 8), reports (n = 9) (including 4 NHS reports), letters to Editors (n = 5), a conference abstract (n = 1) and a website report (n = 1). There was large variation in studies’ aims. Some were concerned with measurement of concordance between preferred and actual place of death, others the evaluation of service redesign on place of death, still others were audits of current preferences to inform service redesign or population studies seeking to inform EOLC policy.

Diverse research methods were used. Patient records (n = 32) were commonly consulted often from Preferred Priorities for Care (PPC) documents (n = 11) (an advance care planning tool designed to encourage the discussion, recording and implementation of patient preferences). Others used questionnaires and surveys (n = 19), interviews (n = 5) or a combination of these methods (n = 2).

Most reports (n = 21) included a range of cancer and non-cancer illnesses. Some (n = 12) did not state the participants’ illnesses, some studied only cancer (n = 8) or specific non-cancer conditions (n = 7) [29–33]. None of the studies of the general population (n = 10) had a specific disease focus.

Most reports were of patient preferences (referred from here on as ‘participants’) (n = 48), of which a minority were proxy reports from family carers (n = 11) or from healthcare professionals (n = 1). Ten were surveys of the general population (referred to from here on as ‘public’).

Data collection was undertaken in varied settings; hospital (n = 10), hospices (n = 8), participants’ homes (n = 5), care homes (n = 2) and in the ‘community’ (GP surgeries or a variety of non-acute settings) (n = 6). Studies were also undertaken in ‘multiple’ settings where participants were asked in either primary and secondary care or where the participant was responding on behalf of a patient (n = 17), or among the general population where location was not relevant (chart category ‘N/A’ (n = 10)).

### Preferences for place of death by participant condition

Fig 4(A) displays preferences for place of death by participants’ main condition. Across all conditions (and none), median preference for home varied only by 9 percentage points, suggesting a broad consensus across all conditions and the public. However, the range of public preferences was much smaller than the range for all other categories. Studies of participants with a variety of different conditions, studies of cancer participants, participants with a non-malignant disease or where the disease focus was not stated each had a range greater than 50 percentage points (respectively 73, 54, 100 and 100 versus 19 percentage points for public).

When missing data were included (Fig 4(B)) the above consensus amongst participants with different health conditions and between participants and the general public disappeared. Only in studies of the general public did median preference for home exceed 50% (median = 62%). Median preference for home for all participants fell; amongst cancer participants to 36%, for multiple conditions to 28%, non-cancer to 42% and for those where the disease focus was not stated to 35%.

### Preferences for place of death by participant role

When considering the roles of contributors, preferences of participants and the general public were broadly similar (Fig 5(A)), with median home preferences of 60% and 63% respectively. However, the range of responses for home was much larger among patients than the public (85 versus 19 percentage points respectively). Studies of patient preferences collected by asking proxies, usually family caregivers (often after the patient’s death), had a high median preference for home of 72%, exceeded only by the one study of healthcare professional proxy perspectives of patient preferences (median 100%). In all but one of the studies of family caregivers, home was the PPOD of at least 58% of respondents; the exception was a study where all respondents chose care home [34].

Fig 5(B) shows preferences by participant role with missing data included. Home preference reported by patients and family members were markedly reduced (to 40% and 27% respectively). Of the 12 studies of family preferences, 9 had large amounts of missing data, ranging from 33 to 72% of all responses. The single study of professional perspectives had no missing data [35]. Patient preferences for home death had a very wide range of 82 percentage points. Public preferences had little reported missing data and median home preference remained high at 62%.

### Preferences for place of death by where participants were asked

Fig 6(A) shows the respondents’ preferences according to the setting in which they were asked. In the two studies where participants were care home residents, all chose care home as their PPOD. Participants at home or in the community tended to prefer death at home (median 81% and 78% respectively). In all but one of the five ‘home’ studies at least 79% of participants chose home [28]. Participants in hospice had lower preferences for home (median 60%) with hospice as the second largest or majority preference in 11 of 12 studies. Those in hospital had the lowest home preferences of all locations (median 50%), excluding care home participants. Participants asked in multiple settings gave home as the most frequent PPOD in all but one study (median 68%). In all general public studies (setting ‘n/a’) home was the PPOD for at least 55% of each study (median 63%).

When missing data were included (Fig 6(B)), median preference for home was greater than 50% in participants in the community, at home or members of the public; for all other groups the median preference for home did not exceed 33%. At least half of studies in hospice, hospital or multiple locations recorded more than 33% of missing data (6/12, 6/11 and 12/18 respectively, where number of missing studies/total number of studies in category). Median preferences for care home remained high amongst studies of care home participants (92%).

### Trends

All three pairs of charts revealed the following trends. When missing data were *excluded* home was the majority PPOD of the study population in 53 of 65 reports. However, missing data accounted for as much as 87% of preferences in one report [29] and for 50% or more of preferences in a further 17 reports. When missing data were *included*, home was the majority PPOD of the study population in only 36 of 65 reports. Studies of the general public reported little missing data and consistently reported home as the most preferred place of death, with preference for home across these studies only ranging by 19 percentage points.

## Discussion

Our review shows that when missing data were excluded the majority of participants preferred to die at home. However, when the large amount of missing data were included in the analysis, it could not be stated that home was known to be where most participants with cancer or other conditions wanted to die. Preferences for place of death appeared to largely reflect where participants were cared for but not necessarily their medical diagnosis. Participant preferences for home seemed to be far more heterogeneous than those expressed by the general public, as demonstrated by the wide range of different values for home preference by participants. Neither the general public nor family caregivers appeared to be accurate proxies for patients’ preferences for place of death.

### Interpretation of findings

In many reports there was a large proportion of missing participants’ preferences. The ‘missing’ preferences are likely to represent preferences that were not asked or not expressed. Preferences may have been missing because participants may not have been given the opportunity to state their preference, and so could reflect the difficulty healthcare professionals have in holding EOLC conversations [36]. Such ‘missing’ preferences could therefore denote unrecorded preferences for death in any setting. Preferences may also be missing because participants did not have a preference to give. Missing preferences like these suggest that participants prioritised other EOLC issues such as pain and symptom management over place of death [5]. Participants may also have been excluded from analysis because they; were undecided about where they wished to die, did not wish to talk about preferences for place of death, were impaired cognitively or physically from communicating their preference or did not have their preference recorded [37]. Regardless of the reasons, the exclusion of ‘missing’ preferences from study reporting inflates the significance of recorded preferences.

Our data shows that it is not known what proportion of cancer patients preferred to die at home due the extent of missing data. This is of consequence given that EOLC provision has historically focused on the needs of cancer patients [1], and current policy rhetoric assumes that home is where most patients wish to die. It may be therefore that preferences do not correlate with this rhetoric.

The variance we found between the preferences of the general public and patients is reported by others [6], and could in part be explained by differences in data collection. For example, information provided about the general public was often drawn from large surveys whilst patient preferences were often collected from patient records. The dissimilarity between public and patient preferences may also in part be attributed to the different meanings given by respondents to questions about PPOD. It is plausible that members of the public asked a hypothetical question[15] concerning a terminal illness may give a different response to a patient who is actually dying. Patient preferences for place of death have been shown to be highly contingent and part of a process rather than absolute *a priori* decisions [9].

Family members’ proxy reports of patients’ PPOD contained a large amount of missing data. One reason for this may partially be due to compromised recall because of the period between bereavement and when participants are asked to take part in research[38]. It may also in part be due to a reluctance of patients to disclose their PPOD to relatives; either because they did not wish to be a burden [39], or because where they died was not a high priority for them[5].

Where participants were asked about their PPOD appeared broadly associated with where they wished to die. We cannot ascertain causation from this association; it may be because participants were in their preferred place of care when asked their preference. The association could also suggest that preferences are contextualised by patients’ experience of care [37] rather than being an isolated choice and therefore may demonstrate that patients prefer familiarity over change at the end of life [40].

### Policy and practice implications

The review has demonstrated a substantial amount of missing data on UK participants’ preferences for place of death. We do not know what locations, if any, these ‘missing’ preferences are for and we should therefore be careful about asserting that the majority of patients wish to die at home.

The extent of missing data has major implications for clinical practice. Some patients may have preferences that are not elicited, calling for sensitive communication skills to encourage them to express their views. Others may not wish to express their preference, or may have no preference and this should be respected [41]. Healthcare professionals, researchers, policy makers and others involved in the care of dying patients need to recognise that not having a preference for place of death is a legitimate opinion which should be recorded in the same way as preferences for specific locations [7]. Including a “missing” preference as a valid category for reporting and analysis would also aid future research.

It was necessary to use basic analytic methods -simple summary statistics of medians—to compare preferences for place of death because much of the available UK evidence of PPOD was not suitable for more sophisticated methods such as meta-analysis. These limitations are seldom explicitly acknowledged in policy.

Whilst general public surveys are valuable in assessing public opinion, they do not appear to reflect dying patient preferences. Likewise, family members appear to be poor proxies for patient PPOD. This has implications for UK health policy which relies heavily on next-of-kin reports, particularly the national survey of bereaved people (“VOICES: Views of Informal Carers—Evaluation Of Services” [42]). The association between where participants were asked their preferences and their PPOD, regardless of the direction of inference, suggests that caution is needed in assuming that home should be the default location for future care for dying patients who are currently being cared for in other settings.

### Limitations and strengths

Our calculations for missing data are limited to ‘reported’ missing data, which is where population and sample sizes are detailed. Public opinion surveys were particularly likely to not report the number of participants who did not state a preference for place of death. In these studies it was not possible to present recalculated preferences to reflect ‘missing’ data, and this may explain some of the difference between the homogeneity of public preferences against the heterogeneity of patient preferences. Likewise some audit studies of PPC documents did not report how many records they viewed which did not have information on PPOD (e.g.[43]). Where studies included only a set number of responses (e.g.[44]), we do not know how many participants were not asked their preferences. For some papers, calculations of preferences including ‘missing’ data are only estimates as only weighted preferences for place of death were reported.

The distribution of “missing” preferences is unknown and it is therefore not possible to speculate on where these participants would have preferred to die. These unknown preferences could represent participants not having a preference at all. Missing preferences may also represent participants having a PPOD other than home, or they could support the policy rhetoric that most dying patients wish to be at home.

When the missing data were included, the proportions of preferences for all locations were reduced. Consequently, of the *known* preferences, the majority of respondents still preferred home over other locations as a place of death, thereby supporting the current policy focus. However, this interpretation overlooks the scale of the missing data.

The heterogeneity of our sample of patient and carer studies may explain the variations of the responses identified, rather than being an inherent feature of these preferences for place of death [6]. The association between where participants were asked about preferences and their PPOD does not imply causality; participants’ location may have already been their PPOD, rather than the experience of where they were asked affecting their PPOD.

The paper builds on the landmark literature review on preferences for place of death of cancer patients by Higginson and Sen Gupta (2000) by considering for the first time ‘missing’ preferences [13]. As outlined above, our findings have direct relevance to policy makers, and for healthcare professionals substantiates anecdotal experience that not all patients want to talk about their preferences or wish to die at home [45].

Whilst the literature review is limited to UK data, the findings are applicable to a broader context. Global life expectancy has continued to increase in recent decades [46] as has the number of adults who have multi-morbidities or a disability at the end of life [47]. Related has been the shift in deaths across the world from communicable to non-communicable diseases [48], meaning that patients are more likely to be chronically ill for an extended period prior to death. As a result of these trends, a preference for place of death is now a meaningful choice for many patients, especially those in the developed world. Critical to understanding these preferences is recognising the nuances involved in PPOD, as demonstrated in the extent of missing preferences found in the review.

## Conclusion

Our review has demonstrated that it is unknown what proportion of UK patients prefers to die at home. Home was a majority preference, but only when missing data were excluded. We found no clear difference between preferences for home and the diagnosis of patients. The homogeneity of the perspectives of the general public or family caregivers correlates poorly with the heterogeneity of patient wishes for PPOD. Preferences may be contextualised by where participants are being cared for when they are asked their preferences. Ultimately, preferences for place of death appear to depend on who is asked the question; what, where, why and when they are asked; and how those without an answer are included.

## Supporting Information

S1 Dataset Dataset for Figs 4–6. (XLSX)

S1 Fig Search terms used for scoping review. (DOCX)

S2 Fig PPOD by data source (A) and including missing data (B). (PDF)

S1 Table Summary of included papers. (DOCX)

S2 Table PRISMA checklist. (DOC)

## Abbreviations

EOLC: end of life care
PPC: Preferred Priorities for Care
PPOD: patients’ preferred place of death
VOICES: Views Of Informal Carers—Evaluation of Services
