# Supplementary material for: Do Patients Want to Die at Home? A Systematic Review of the UK Literature, Focused on Missing Preferences for Place of Death
Source: PLoS One. 2015 Nov 10;10(11):e0142723. doi: 10.1371/journal.pone.0142723 (PMC4640665; doi:10.1371/journal.pone.0142723)
Supplement: S1 Fig — (DOCX) [file pone.0142723.s002.docx]

**Search strategy used for scoping review of Embase**

| Location | Date | Search terms | Results |
| --- | --- | --- | --- |
| Embase | 25/02/14 | (("place of death" OR "place of care") AND (pref* OR wish* OR choice OR decision*)).ti,ab [Limit to: Publication Year 2000-2014] | 409 |

The search was carried out for papers published between 1999 and March 2014 in:

Medline

EMBASE

PsycINFO

CINAHL

British Nursing Index

AMED

ASSIA

Scopus

ISI Web of Knowledge

Health Management Information Consortium

.
