# Supplementary figures and images for: Do Patients Want to Die at Home? A Systematic Review of the UK Literature, Focused on Missing Preferences for Place of Death
Source: PLoS One. 2015 Nov 10;10(11):e0142723. doi: 10.1371/journal.pone.0142723 (PMC4640665; doi:10.1371/journal.pone.0142723)

Preference for place of death by data source

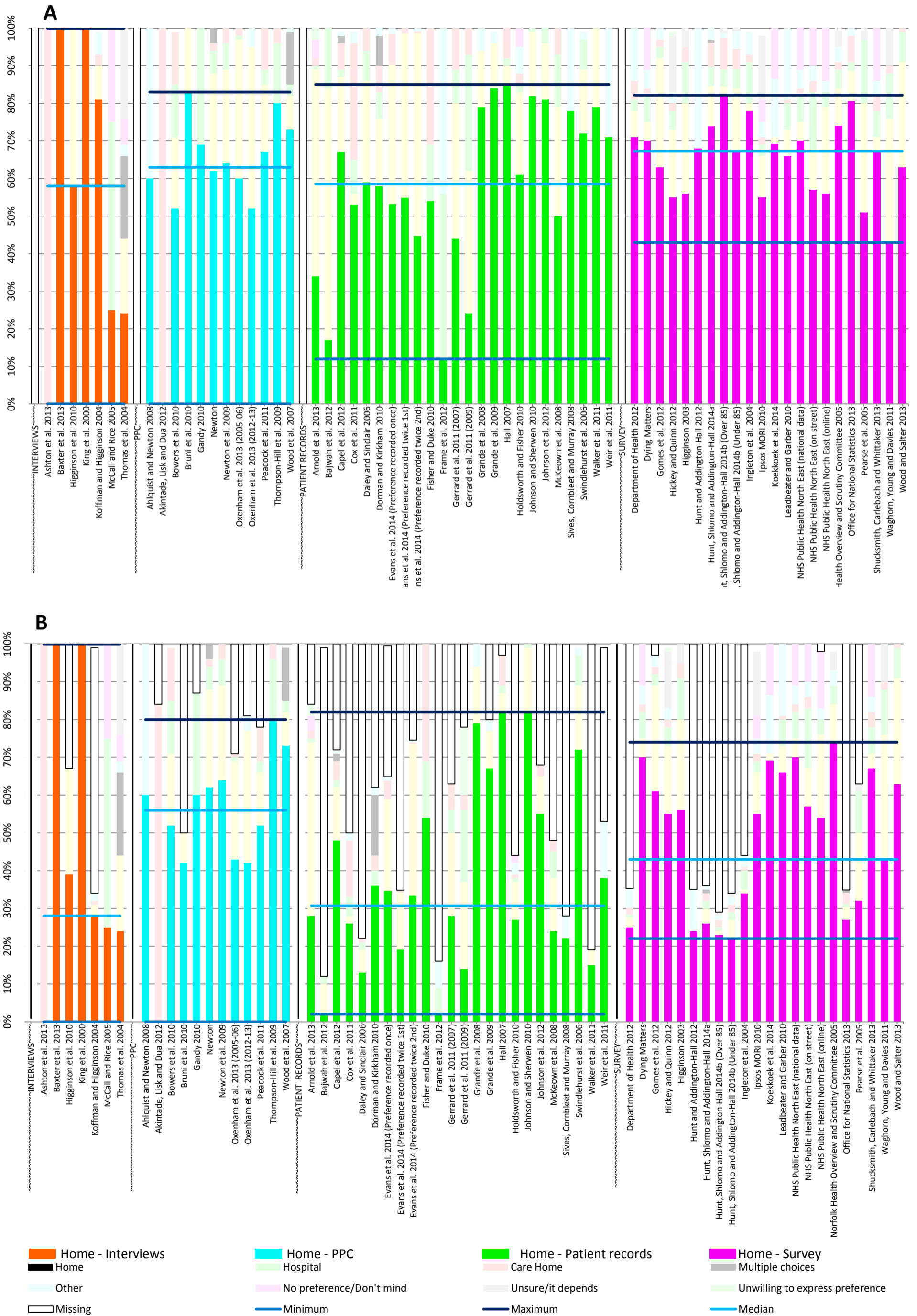

Supplement: S2 Fig — (PDF) [file pone.0142723.s003.pdf]
