# Supplementary material for: Do Patients Want to Die at Home? A Systematic Review of the UK Literature, Focused on Missing Preferences for Place of Death
Source: PLoS One. 2015 Nov 10;10(11):e0142723. doi: 10.1371/journal.pone.0142723 (PMC4640665; doi:10.1371/journal.pone.0142723)
Supplement: S1 Table — (DOCX) [file pone.0142723.s004.docx]

| **Study** | **Report type** | **Study description** | **Setting/**  **Recruitment sites** | **Disease focus** | **Population** | **Data source** | **Date** | **Key findings as reported in text** | **WoE A** | **WoE B** | **WoE C** | **WoE Mean** |
| --- | --- | --- | --- | --- | --- | --- | --- | --- | --- | --- | --- | --- |
| **Ahlquist, and Newton (2008) [1]** | Poster | Evaluation of impact of Preferred place of care (PPC) documents. Patients cared for within South Essex Cancer Network with PPC documents | South Essex Cancer network | (Assume) Cancer | Patients | PPC document | Oct 2005 - Sept 2007 | Total participants n=182.  60% preferred home | 1 | 1 | 1 | 1 |
| **Akintade, Lisk et al. 2012. [2]** | Poster | Advance care planning (ACP) project in nursing homes. | nursing home |  | Patients and next of kin | ACP form |  | Total participants n=25, completed forms of n= 21.  100% Nursing home | 2 | 2 | 2 | 2 |
| **Arnold, Finucane, et al. (2013). [3]** | Research paper | Assess preferred place of death (ppd) for patients referred to a specialist palliative care service, including why PPD is not recorded and difference between hospice inpatients and non-inpatient preferences and to explore congruence between preferred and actual place of death | Marie Curie Hospice Edinburgh | 94% patients had cancer | Patients and for a few next of kin/carers | patient records | 2009-2010 | Total participants n= 1127.  60% hospice, 37% home, 2% care home, 1% hospital.(23% didn't have PPD recorded because; 31% concern hadn't known patient long enough, 21% undecided or place unimportant, 19% healthcare professional (HCP) concern cause patient distress, 15% unwilling to talk about it, 12% cognitive impairment/inability to communicate/multiple reasons, 1% HCP felt too early to ask). | 2 | 2 | 2 | 2 |
| **Ashton, Roe et al. (2013). [4]** | Research paper | Family carers' views on how advanced care planning contributed to end of life care for people with advanced dementia (n=6) | Care home | Dementia | family caregivers | semi-structured interviews |  | Total participants n=6.  100% Care home. | 2 | 2 | 1 | 2 |
| **Bajwah, Higginson, et al. 2012.[5]** | Research paper | Patient group needs assessment. A review of deceased patients notes for recorded preferred place of care and death. | Royal Brompton Hospital and King's College Hospital, London | Idiopathic fibrotic interstitial lung disease | Patients | Case notes | Jan 2009 - May 2010 | Total participants n=45.  2% Home (n=1), 8% Hospice (n=4), 2% hospital (n=1), 87% Not documented (n=39). Of PPC 11% Home (n=5) , 4% hospice (n=2) , 2% hospital (n=1), 82% not documented (n=37) | 3 | 2 | 2 | 2 |
| **Baxter, Baird et al., 2013 [6]** | Research paper | Study exploring carer and health professional experiences of end of life care of motor neurone disease patients using non-invasive ventilation. | Not stated. | Motor Neurone Disease | Family carers and health care professionals | In-depth Interviews | May 2010- April 2012 | 100% Home (n=10) | 2 | 2 | 1 | 2 |
| **Bowers, Roderick et al. 2010. [7]** | Research paper | Evaluation of service redesign on patient care. Audit of recorded patient preferences. | GP practice in Cambridgeshire |  | Patients | PPC document | 6 month period | Total participants n=21. Home (n=11), hospice (n=4), nursing home (n=3), hospital (n=3). | 1 | 1 | 1 | 1 |
| **Bruni, Plunkett et al. 2010. [8]** | Poster | Audit of PPC document use and concordance. | Farleigh hospice, Chelmsford | Chronic Obstructive Pulmonary Disease | Patients | PPC document | May 2008 - July 2009 | Total participants n =12.  home (n=5), hospice (n=1) not known (n=6) | 2 | 2 | 2 | 2 |
| **Capel, Gazi et al. 2012 [9]** | Research paper | Study of concordance for all deceased patients who known to a specialist palliative care service. | George Thomas Hospice Care, Cardiff | 93% cancer | Patients | Case notes | Jan 2009 - 31 Dec 2010 | Total participants n =788.  Home (n=380), hospice (n=114), nursing home (n=36), hospital (n=14), residential home (n=5), prison (n=1), abroad (n=1), home or hospice (n=11), home or hospital (n=2), hospital or hospice (n=1), unwilling to express preference (n=224). | 3 | 2 | 3 | 3 |
| **Cox, Moghaddam et al. 2011. [10]** | Research paper | Audit of practice to discuss and record preferences. Random sample of deceased patients from regional cancer network. | Hospital Specialist Palliative Care service, GP practice, Heart failure community matron service, nursing home | Cancer (n=36), heart failure (n=24), no recorded diagnosis (n=5). | Patients and carers | Case notes | Jan 05 - Dec 06 | Documented preference n=32, of total participants n=65.  GP Practice (10/19):  80% Home (n=8), 10% hospice (n=1), 10% care home (n=1).  Heart Failure Community Matrons (3/15):  67% home (n=2), 33% care home (n=1)  Hospital palliative care service (11/15): home (n=7), 64% hospital (n=2 18%), 9% care home (n=1) 9% patient preferred home, carer hospital (n=1).  13% Nursing Care Home (8/16): hospital (n=1), 88% care home (n=7).  In total (32/65):  53% home (n=17), 9% hospital (n=3), 3% hospice (n=1), 31% care home (n=10), 3% patient home, carer hospital (n=1). | 3 | 2 | 2 | 2 |
| **Daley and Sinclair 2006. [11]** | Letter to Editor | Study of concordance. Review of records of deceased patients receiving specialist palliative care services. | Bradford and Airedale, West Yorkshire. | Not stated | Patients | shared patient electronic record system | Oct 2003 - Sept 2005 | 1072 patients of whom 243 had PPD recorded.  59% Home (n=144), 27% Hospice (n=65), 8% Hospital (n=20), Nursing or residential home 6% (n=14) | 2 | 2 | 2 | 2 |
| **Department of Health (2012) [12] AND Office for National Statistics (2012) [13]** | Report | National survey of bereaved people evaluating end of life services. | England | none | Bereaved informal carers (4-11months post bereavement) | VOICES survey | Autumn 2011 | Total participants answering question n=19,888.  71% Home, 3% Hospital, 5% Care Home 7% Hospice 14% somewhere else (52%, Patients hadn't expressed a preference 5% Unsure if had a preference) | 2 | 2 | 1 | 2 |
| **Dorman and Kirkham 2010. [14]** | Poster | Audit of practice in recording patient preferences and concordance of deceased specialist palliative care patients. | Dorset network specialist palliative care group. | Not stated | Patients | PPC document | Dec 2008 - Feb 2009 | Total participants n =470.  62% documented PPC.  36% home, 11% specialist palliative care (SPC) unit or hospice, 3% either home or SPC unit, 4% care home, 4% hospital, 2% combination of the above, 2% other, 38% unknown. | 2 | 2 | 2 | 2 |
| **Dying Matters [15]** | Website | Study of public attitudes and preferences. | England, Scotland and Wales | None | Population | Interview survey | July-Sept 2009 | Total participants n= 1,375.  70% home | 2 | 2 | 1 | 2 |
| **Evans, Finucane et al. 2014 [16]** | Research paper | Retrospective case note review of patients who died under the care of a specialist palliative care service to establish whether there are changes to patient preferences. | Marie Curie Hospice Edinburgh |  | Patients | Case notes | 6 month period in 2012 | Total participants n=299  Patients whose preferences were recorded once n=95:  35% Home, 24% Hospice, 5% Care Home, 1% Relatives, 35% Unknown  Participants whose preference was recorded twice n=204.  First recorded preference:  Home (n=39), Hospice (n=31), Care Home (n=1), Relatives (n=0), Unknown (n=133)  Second recorded preference:  Home (n=68), Hospice (n=82), Care Home (n=1), Relatives (n=1), Unknown (n=52) | 2 | 2 | 3 | 2 |
| **Fisher and Duke 2010. [17]** | Research paper | Audit of referrals patients referred for to hospital discharge team for end of life care (EOLC) discharge. | Southampton hospital | Cancer and non-cancer | Patients | medical records or asked patients | May-July 2009 | Total participants n=39.  54% Home (n=21), 31% Nursing home (n=12), 15% Hospital (n=6) | 1 | 2 | 1 | 1 |
| **Frame, Ring, et al. 2012. [18]** | Poster | Baseline audit of current practice in hospital of advance care planning, EOLC pathways and achievement of PPC and PPD. Retrospective case note audit of 163 consecutive adult deaths. | 3 London acute hospitals | All. 33% cancer | Patients | Case notes | 2010 | Total participants n=163 patients. PPC: 52% Hospital (n=13), 28% home (n=7). PPD 44% Hospital (n=11), 12% Home (n=3) | 1 | 1 | 2 | 1 |
| **Gandy, 2010. [19]** | Research paper | Economic analysis of the cost of providing care to dementia patients. | Four care homes Greater Manchester. | Dementia | Patients | Deceased patient records | Oct 2006-Set 2007 | Total participants n=30.  60% home (n =18), 27% hospital (n=8), 13% no preference recorded (n=4). | 2 | 2 | 2 | 2 |
| **Gerrard, Campbell, et al. 2011. [20]** | Research paper | Audit of congruence of deceased patients referred to hospital palliative care team. | St George's Healthcare NHS Trust, London | Between 2/3rd (2007) and 3/4 (2009) patients had cancer | Patient or carer | Case note review and in 2009 also 'specific internal documentation.' | Jan -June 2007, April -Sept 2009 | 2007: Total participants n=236 of which n=149 patients had recorded preference.  44% Home (n=66), 36% hospice (n=52), 9% hospital (n=14), 11% nursing home (n=17). Total number of preferences 149/236.  2009: Total participants n=275 of which n=166 patients had recorded preference.  24% Home (n=39), 38% hospice (n=63), 31% hospital (n=52), 7% nursing home (n=12). Total preferences 166/275. 49 patients refused to discuss PPC. | 2 | 2 | 2 | 2 |
| **Gomes, Higginson, et al. 2012. [21] AND Gomes, Calanzani, et al. 2011.[22]** | Report AND Research paper | European study of patient preferences for place of death. | All regions in England. | None | Population | Telephone survey | May - Dec 2010 | 1351 randomly selected adults.  Own home 63% (n=829), home of a relative or friend (n=15)1.1%, hospice or palliative care unit 29% (n=381), hospital - but not palliative care unit 3.2% (n=42), care home 2% (n=26), Elsewhere 1.7% (n=23) | 2 | 2 | 2 | 2 |
| **Grande and Ewing 2008**  **[23]** | Letter to Editor | Study of patient and carer preferences for death at home. | 3 hospice at home services | not stated | Patient and carer (Patient responses only reported in Table 2). | Patient records | 4 month period | Total patient and carer preferences n= 255.  79% Home (n=201), 15% hospice (n=39). | 2 | 2 | 2 | 2 |
| **Grande and Ewing 2009. [24]** | Research paper | Study of impact on congruence on carers' bereavement outcomes of deceased patients using one of five ‘hospice at home’ services. Review of patient records and carer interviews. | 5 hospice at home services | Cancer and non-cancer | Patient and carer (Patient responses only reported in Table 2). | Electronic data record | 4 month period | Total participants n= 216, patients recorded preferences n=172  84% Home (n=145), 12% hospice (n=21). 97 carers expressed preferences (45% of total). 70% home (n=68), 27% hospice (n=26) | 2 | 2 | 2 | 2 |
| **Hall 2007.[25]** | Research paper | Review of audit tools completed by nurses to assess patient need and measure congruence. | Easington, Co. Durham. | 90/108 had cancer. rest had variety of conditions | Patients | Nurse-completed audit tool | Autumn 2005- Spring 2006 | Total participants n= 108.  Home (n=89), hospice (n=5), care home (n=9), hospital (n=2), Unknown (n=3) | 1 | 1 | 1 | 1 |
| **Hickey and Quinn 2012. [26]** | Research paper | Awareness-raising campaign in South West Essex about end of life care planning. | Two main town centres in South West Essex. | Population | Population | Survey at public awareness roadshow | March 2010 | Total participants n=304.  63% home, 21% hospice, 6% hospital, 2% nursing home, 0.5% residential home, 17% unable to decide, 4% other. 1% didn't respond. | 2 | 2 | 2 | 2 |
| **Higginson (2003) [27]** | Research paper | National survey to assess priorities and preferences for end of life care. | England, Wales, Scotland | Population | Population | Telephone survey | April 2002 | Total participants n=1000.  Home 56%, Hospice 25%, Hospital 11%, Nursing/Residential home 4%. | 2 | 2 | 1 | 2 |
| **Higginson, Hall *et al.*, 2010a [28] AND Higginson, Hall, et al., 2010b [29]** | Poster AND Letter to Editor | Study to examine changes in relatives’ preferences for place of death. | Palliative care service of a London Hospital | Cancer | Bereaved relatives | interviews (n=8) or postal questionnaire (n=10) |  | Total participants n=18.  Home (n=7), hospice (n=5) | 1 | 2 | 1 | 1 |
| **Holdsworth and Fisher 2010 [30]** | Research paper | Explore recording rate of preferences and the congruence between preferred and actual place of death. | 3 hospices in South East England | Majority of patients had cancer | Patients | Hospice records | 6 month period | Total participants n=298.  27% Home (n=80), 10% hospice (n=29), 1% hospital (n=2), 1% care home (n=4), 3% Other (n=9), 2% no preference (n=7), 56% missing (n=167). | 3 | 2 | 3 | 3 |
| **Hunt and Addington-Hall 2012 [31]** | Oral presentation abstract | To explore end of life care preferences and determine predictors of achieving preferred place death (home death and hospital death). | Two health districts in UK | All | Bereaved relatives | self-completion post-bereavement survey | April 2010 - 2011 | Total participant n=473.  68% home. | 1 | 2 | 1 | 1 |
| **Hunt, Shlomo et al. 2014a [32]** | Research paper | Explore reported preferences for place of death and experiences of care in a population based sample of deaths from all causes. | Two health districts in England | None | Bereaved informal carers | VOICES Short Form Survey | October 2009-2010 | Total participants n= 1142.  “Did he or she ever say where they would like to die?”  36% Yes (n=412) 64% No (n=741)  Where did he say he would like to die?  74% Home (n=296), 11% hospice (n=43), 5% hospital (n=21), 7% care home (n=25), 3% somewhere else (n=13), 1% Changed mind (n=3). | 2 | 2 | 1 | 2 |
| **Hunt, Shlomo et al. 2014b [33]** | Research paper | To explore experiences of EOLC among the oldest old and determine their reported preferences for place of death. Compared oldest old (over 85 year old preferences with under 85 year olds. | Two English health districts | All (cancer 15.8%) | Bereaved relatives | self-completion post-bereavement survey | April 2010 - 2011 | Total participants n=473.  Over 85 weighted n= 657.6: 82.2% Home, 4.6% Hospital, 8.3% Care Home, 2.3% Hospice, 1.4% changed mind, 1.2% Somewhere else.  Under 85 years old weighted n = 714.1: 67.5% Home, 5.2% Hospital, 5.3% Care Home, 16.5% Hospice, 0.8% Changed mind, 4.8% Somewhere else. | 2 | 2 | 1 | 2 |
| **Ingleton, Morgan, et al., 2004 [34]** | Research paper | Study of satisfaction with end of life care service in the last year of life. | Powys, Wales | Cancer | Informal carers | Modified VOICES survey | 1999-2001 | Total participants n=301.  “Did they say where they wanted to die?” 50% No (n=151), 3% Don't know (n=10), 3% NA/Missing/other (n=8).  “If yes, where was that place?”  34% Home (n=103), 4% Community Hospital (n=12), 1% older people's or nursing home (n= 3), 1% Hospice (n=3) 1% general hospital (n=3), other (n=8). | 2 | 2 | 2 | 2 |
| **Ipsos MORI (2010) [35]** | Report | Survey of East of England designed to raise awareness in the population and inform service planning. | Population | None | Population | Survey | March - July 2010 | Total participants n=693.  55% Home, 2% care home (either residential or nursing), 3% hospital, 11% hospice, 4% somewhere else, 6% don't mind, 17% it depends | 2 | 2 | 2 | 2 |
| **Johnson and Sherwen (2010) [36]** | Research paper | Evaluation of the use of Preferred Priorities for Care documents on place of death by reviewing first 100 patients who had completed PPC and subsequently died. | NHS West Essex | 83% cancer | Patients | PPC document | July 2008 - Oct 2009 | Total participants n=100.  1% Hospital, Hospice 17%, Home 82%. | 1 | 1 | 1 | 1 |
| **Johnson, Nunn *et al.*, 2012. [37]** | Research paper | Needs assessment and evaluation of service for heart failure patients served by two teams. Prospective data gathered by heart failure nurse specialists of deceased patients. | Bradford/Airedale and Scarborough | Heart failure | Patients | Data collection sheet | Jan- Dec 2009 (Bradford/Airedale) April -March 2010 (Scarborough) | Total participants n=126.  Home (n=69), hospice (n=12), hospital (n=4). Approximately as read from bar chart | 1 | 2 | 2 | 2 |
| **King, Mackenzie et al. 2000[38]** | Research paper | Evaluation of a hospice rapid-response service. Sample drawn from patients referred to hospice service. | Highland Hospice, Scottish Highlands |  | Patients | Hospital, hospice local statistical records, interviews, focus groups, questionnaires. | April 1998- March 1999 | Total participants n=17  All expressed wish to die at home | 2 | 2 | 1 | 2 |
| **Koekkoek, Dirven et al. 2014 [39]** | Research paper | European comparative study of end of life care processes for high-grade glioma patients, focused on The Netherlands, Austria and the UK. | Western General Hospital, Edinburgh | High-grade glioma | Bereaved relatives | Questionnaire | 2012 | Total participants n=52  69% Home (n=36), 2% Hospice (n=1), 0 Hospital, 0 Nursing/care home, 14% No preference (n=7), 15% Other (n=8) | 2 | 2 | 3 | 2 |
| **Koffman and Higginson 2004 [40]** | Research paper | Study to explore the preference for place of death of black Caribbean population living in the United Kingdom as compared to native-born patients with advanced disease. | 3 inner-London Boroughs | 68% cancer | Next-of-kin/friend | Interviews with carers | 13 months | Total participants n=100  Caribbean descent n=50  85% Home (n=17), 5% hospice (n=1), 10% return to Jamaica (n=2).  White British n=50  75% Home (n=9), 17% hospice (n=2), 8% care home (n=1). | 2 | 2 | 2 | 2 |
| **Leadbeater and Garber 2010 [41]** | Report | YouGov panel representative of all GB adults, designed to measure public attitudes towards death and dying. | Online | All | Population | Survey | Sept 2010 | Total participants n=2127.  66% home 1% care home 7% hospital, 7% hospice. | 2 | 2 | 1 | 2 |
| **McCall and Rice 2005 [42]** | Research paper | Exploring factors about place of care with terminally ill patients. N=8 | West Highland, Scotland | Cancer | Terminally ill patients | semi-structured interviews |  | Total participants n=8.  Hospital (n=4), Home (n=2), No overwhelming preference (n=2). | 2 | 3 | 3 | 3 |
| **McKeown, Agar et al. 2008 [43]** | Research paper | Retrospective audit to assess referral practices for patients with End-Stage Renal Failure to Liverpool University Hospital Specialist Palliative care. | Royal Liverpool University Hospital palliative care team | End-stage renal failure | Patients | Case notes then audit form | March - April 2006 | Total participants n=29.  PPC documented in n=14.  50% Home (n=7), 29% hospice (n=4), 14% hospital (n=2), 7% nursing home (n=1). | 2 | 2 | 2 | 2 |
| **Newton [44]** | NHS Report | Review of the introduction of a pilot of PPC use in South Essex. | Acute and community settings across South Essex. | Majority cancer. | Patients | PPC (adapted for local use) | Oct 2005-Oct 2006 | Total participants n=94  62% home, 25.5% hospice, 6.2% hospital, 2.2% nursing home, 4.3% 2 options | 1 | 1 | 1 | 1 |
| **Newton, Clark et al. 2009 [45]** | Research paper | Evaluation of the introduction of an advanced care plan into community and inpatient settings. | Acute and community settings across South Essex. | majority cancer patients | Patients | adjusted PPC document, | Oct 05-Sept 07 | Total participants=182.  64% home, 7% hospital, 25% hospice, 4% care home | 1 | 1 | 1 | 1 |
| **NHS Public Health North East 2010[46]** | NHS Report | Public consultation on ‘what makes a good death’. | On-street survey, North East.  Online survey, North East.  National data, UK. | All | Population | On street survey, online survey, national survey | On street survey- Oct 2009, online survey Oct-Dec2009, National survey July-Sept 2009 | National (total n=1290):  70% home, 7% hospital, 5% hospice, 1% nursing home, 1% care home, 5% somewhere else, 12% don't mind.  On street (total n=1117): home 57%, hospital 16%, hospice 10%, nursing home 2%, care home 1%, somewhere else 4%, don't mind 11% Online (n=1267): home 56%, 4% hospital, 23% hospice, 3% somewhere else, 15% don't mind | 2 | 2 | 2 | 2 |
| **Norfolk Health Overview and Scrutiny Committee (2005) [47]** | NHS Report | Review of palliative care services including a consultation of the general public on attitudes, experiences and expectations of end of life care. | Norfolk County and Waveney District | Population | Population (Norfolk and Waveney Citizen's Panel members) | Self-completion survey |  | Total participants n= 524  74% own home, 10% hospice, 6% don't know, 5% main hospital, 4%, elsewhere, 3% nursing home, 2% community hospital, 1% someone else's home.  *** Figures are approximate and are derived from graph | 1 | 2 | 1 | 1 |
| **Office for National Statistics (2013)[48]** | Report | National survey of bereaved people evaluating end of life services. | England | none | Bereaved informal carers (4-11months post bereavement) | VOICES survey | 15 week period. | Total survey respondents n= 22,635. For question on preferences for place of death n=21,973.  81% Home, 8% hospice, 2% hospital, 7% care home, 2% somewhere else | 2 | 2 | 1 | 2 |
| **Oxenham, Finucane et al. 2013[49]** | Research paper | Hospice PPD audit and re-audit 7 years later following changes to practice to improve identification, communication and achievement of preferred place of death. | Marie Curie Hospice Edinburgh |  | Patients | Patient records | 2005-2006 (first audit) and 2012-2013 (second audit) | 2005-2006 (n=164):  60% home, 37% hospice, 2% nursing home, 1% hospital.  2012-2013 (n=196):  52% home, 43% hospice, 5% other locations.  19% had no recorded preference (n=37). | 2 | 2 | 2 | 2 |
| **Peacock, Riches et al. 2011[50]** | Poster | Audit of completion of patient advanced care plans. | Royal Derby Hospital, Derby | Colorectal cancer | Patients | ‘Gold Record' care plan | May 2009 – Jan 2010 | Total participants n=45.  67% home (n=30), 5% care home (n=11), 18% hospice (n=8), 4% hospital (n=2) | 2 | 2 | 2 | 2 |
| **Pearse, Saxby et al. 2005 [51]** | Letter to Editor | Looking at why patients often did not achieved preferred place of death. Considered 100 consecutive terminally ill patients referred to palliative care team. | Leeds Teaching Hospital Palliative Care Team | 90% cancer patients | Patients | Patients were asked their PPD |  | Total participants n = 100.  51% home, 29% hospice, 17% hospital, 3% unsure | 2 | 3 | 2 | 2 |
| **Shucksmith, Carlebach et al. 2013[52]** | Report | 2012 British Social attitudes survey, a representative national survey of adults over the age of 18. response rate was 53%, | National survey | none | Public | National survey | June-Nov 2012 | Total participants n=3248.  67% home , 7% hospital, 4% hospice, 4% somewhere else, 14% 'do not mind' | 2 | 2 | 1 | 2 |
| **Sives, Cornbleet et al. 2008 [53]** | Poster | Audit of proportion of patients referred to a UK hospice specialist community team who have their PPC documented. | Hospice |  | Patients | Case note review | July 2006 - June 2007 | Total participants n=583 with PPD data available for 28% (n=162).  78% home. | 2 | 2 | 2 | 2 |
| **Swindlehurst, Walton et al. 2006[54]** | NHS Report | Audit of practice adherence to Gold Standards Framework (GSF) register. | GP practices in Shropshire County and Telford and Wrekin PCT. | Mainly cancer | Not stated | Patient records | April-Sept 2005 | Total participants n=20.  Home (n=144), hospice (n=34), community hospital (n=9), acute hospital (n=9), other (n=5). | 1 | 1 | 1 | 1 |
| **Thomas, Morris et al. 2004.[55]** | Research paper | Qualitative study to explore terminally ill patient and their informal carers’ attitudes to place of death. | Morecambe Bay. Patients referred to researchers by palliative care professionals | Cancer | Patients | Interviews with patients | 2000-2002 | Total participants n= 41.  24% Home (n=10), 20% Hospice (n=8), 22% Home or hospice (n=9), Hospital 0, 3% Other (n=1), 24% Not decided (n=10), 7% No preference (n=3). | 3 | 3 | 3 | 3 |
| **Thompson-Hill, Hookey et al. 2009[56]** | Research paper | Audit of ‘the supportive care plan’ to assess documentation of preferred place of care and patient and family understanding of patients’ illness. | Acute hospital |  | Patients | Supportive Care Plan (similar to PPC) | From April 2008 | Total participants n= 25.  4% Residential home (n=1), 16% community hospital (n=4), 80% home (n=20) | 1 | 1 | 1 | 1 |
| **Waghorn, Young et al. 2011[57]** | Research paper | To explore the relative importance of place of death to cancer in-patients and out-patients. | Royal Marsden Hospital | Cancer | Patients | Questionnaire |  | Total participants n= 120.  Home (n=51), nursing home (n=2), hospice( n=39), oncology centre (n=12), other hospital (n=2), unsure (n=13), no answer (n=1) | 3 | 3 | 3 | 3 |
| **Walker, Read et al. 2011[58]** | Research paper | Audit of use of PPC tool and documentation of conversations relating to preferred place of death using a random sample of notes. | Midlands hospice |  | Patients | Case notes | Jan 2008, Jan2009, Jan 2010 | Total participants n= 150.  79% Home, 21% hospice. | 3 | 2 | 2 | 2 |
| **Weir, Magowan et al. 2011 [59]** | Poster | Audit of palliative care given to patients to inform service development. | Altnagelvin Hospital, Derry | lung cancer | Patients | Asked during final admission | 2010 | Total participant n =26, of which n=14 stated PPD.  Home (n=10). | 1 | 2 | 1 | 1 |
| **Wood and Salter 2013[60]** | Research paper | Report on end of life priorities, focusing on preferences for place of death. Includes a public survey on outcomes participants value at the end of life care | UK | none | Public | Survey |  | Total participant n = 2,038.  63% home, 28% hospice, 8% hospital, 1% care home | 3 | 2 | 2 | 2 |
| **Wood, Storey et al. 2007 [61]** | Letter to Editor | Retrospective analysis of first 100 completed and returned assessments of patients’ end of life care preferences using preferred place of care tool. | North west England, two NHS primary care trusts |  | Patients | PPC document | Feb 03 -Feb 05 | Total participants n=100.  73% home, 12% home or hospice, 9% hospice, 2% community hospital, 1% hospital, 1% home or community hospital, 1% home patient/hospice family, 1% home or hospital or hospice | 2 | 1 | 1 | 1 |

1. Ahlquist PY, Newton J. How effective is the preferred place of care document? 7th Palliative Care Congress; 29th April – 1st May 2008; Glasgow, Scotland. Palliative Medicine: Palliative Medicine; 2008. p. 582.

2. Akintade O, Lisk R, Dua R. Advance care planning in a nursing home. 8th Congress of the European Union Geriatric Medicine Society; 26-28 September 2012; Brussel, Belgium: European Geriatric Medicine; 2012. p. S44.

3. Arnold E, Finucane AM, Oxenham D. Preferred place of death for patients referred to a specialist palliative care service. BMJ Support Palliat Care. 2013:10.1136/bmjspcare-2012-000338. doi: 10.1136/bmjspcare-2012-000338.

4. Ashton S, Roe B, Jack B, McClelland B. Advance care planning: families’ views. JDC. 2013;21(5):28-31.

5. Bajwah S, Higginson IJ, Ross JR, Wells AU, Birring SS, Patel A, et al. Specialist Palliative Care is More Than Drugs: A Retrospective Study of ILD Patients. Lung. 2012;190(2):215-20. doi: 10.1007/s00408-011-9355-7. PubMed PMID: WOS:000302146900011.

6. Baxter SK, Baird WO, Thompson S, Bianchi SM, Walters SJ, Lee E, et al. The use of non-invasive ventilation at end of life in patients with motor neurone disease: a qualitative exploration of family carer and health professional experiences. Palliat Med. 2013;27:516-23. doi: 10.1177/0269216313478449. PubMed PMID: 23462702.

7. Bowers B, Roderick S, Arnold S. Improving integrated team working to support people to die in the place of their choice. Nurs Times. 2010;106(32):14-6. PubMed PMID: MEDLINE:20879639.

8. Bruni C, Plunkett C, Kroeber T. Advanced COPD: is preferred place of care a reality in Mid Essex? 8th Palliative Care Congress; 10-12th March 2010; Bournemouth, UK: Palliative Medicine 2010. p. 226.

9. Capel M, Gazi T, Vout L, Wilson N, Finlay I. Where do patients known to a community palliative care service die? BMJ Support Palliat Care. 2012;2(1):43-7.

10. Cox K, Moghaddam N, Almack K, Pollock K, Seymour J. Is it recorded in the notes? Documentation of end-of-life care and preferred place to die discussions in the final weeks of life. BMC Palliat Care. 2011;10(1):doi: 10.1186/472-684X-10-18. doi: 10.1186/1472-684X-10-18.

11. Daley A, Sinclair K. Recording and auditing preferred place of death. Palliat Med. 2006;20(6):637-8. doi: 10.1177/0269216306070279. PubMed PMID: WOS:000241278900011.

12. Department of Health. First national VOICES survey of bereaved people: key findings report. <http://www.dh.gov.uk/publications:> 2012.

13. Office for National Statistics. National Bereavement Survey (VOICES) 2011. <http://www.ons.gov.uk/ons/rel/subnational-health1/national-bereavement-survey--voices-/2011/national-bereavement-survey--voices---2011.html2012>.

14. Dorman S, Kirkham S. Audit of preferred place of care at end of life. 8th Palliative Care Congress; 10-12th March 2010; Bournemouth, UK. Palliative Medicine: Palliative Medicine; 2010. p. 202-52.

15. Dying Matters. Survey reveals our reluctance to discuss own death. Available from: <http://www.dyingmatters.org/page/survey-reveals-our-reluctance-discuss-own-death>.

16. Evans R, Finucane A, Vanhegan L, Arnold E, Oxenham D. Do place-of-death preferences for patients receiving specialist palliative care change over time? Int J Palliat Nurs. 2014;20(12):579-83. Epub 2014/12/20. doi: 10.12968/ijpn.2014.20.12.579. PubMed PMID: 25526286.

17. Fisher S, Duke S. Preferred place of care at the end of life for people in hospitals. End of Life Care. 2010;4(2):35-41.

18. Frame KL, Ring F, Howard D, Urch C. Current End of Life Care Practices in an Acute Hospital Setting - Important Lessons from an Audit. 7th World Research Congress of the European Association for Palliative Care (EAPC); 7-9 June 2012; Trondheim, Norway: Palliative Medicine; 2012.

19. Gandy R. Economic appraisal of an end-of-life care training initiative for care homes with dementia patients. J Care Serv Manag. 2010;4(4):321-30. doi: 10.1179/175016810X12773688140464.

20. Gerrard R, Campbell J, Minton O, Moback B, Skinner C, McGowan C, et al. Achieving the preferred place of care for hospitalized patients at the end of life. Palliat Med. 2011;25(4):333-6.

21. Gomes B, Higginson I, Calanzani N, Cohen J, Deliens L, Daveson B, et al. Preferences for place of death if faced with advanced cancer: a population survey in England, Flanders, Germany, Italy, the Netherlands, Portugal and Spain. Ann Oncol. 2012;23(8):2006-15.

22. Gomes B, Calanzani N, Higginson I. Local Preferences and Place of Death in Regions within England 2010. London: Cicely Saunders International, 2011.

23. Grande G, Ewing G. Death at home unlikely if informal carers prefer otherwise: implications for policy. Palliat Med. 2008;8:971-2.

24. Grande G, Ewing G. Informal carer bereavement outcome: relation to quality of end of life support and achievement of preferred place of death. Palliat Med. 2009;23(3):248-56.

25. Hall S. Assessing for preferred place of care. Journal of Community Nursing. 2007;21(12):4. PubMed PMID: 2009741009. Language: English. Entry Date: 20080208. Revision Date: 20091218. Publication Type: journal article.

26. Hickey D, Quinn S. ‘I don't want to talk about it.’Raising public awareness of end-of-life care planning in your locality. Int J Palliat Nurs. 2012;18(5):241-7.

27. Higginson I. Priorities and preferences for end of life care in England, Wales and Scotland. London: National Council for Hospice and Specialist Palliative Care Services; 2003.

28. Higginson IJ, Hall S, Koffman J, Riley J, Gomes B. How stable are preferences for place of death? Findings from a pilot study. 6th Research Congress of the EAPC; June 10-12 2010; Glasgow, UK. Palliative Medicine: Palliative Medicine; 2010. p. S188.

29. Higginson IJ, Hall S, Koffman J, Riley J, Gomes B. Time to get it right: are preferences for place of death more stable than we think? Palliat Med. 2010;24(3):352-3. doi: 10.1177/0269216309360489. PubMed PMID: WOS:000276382100014.

30. Holdsworth L, Fisher S. A retrospective analysis of preferred and actual place of death for hospice patients. Int J Palliat Nurs. 2010;16(9):424-31.

31. Hunt KJ, Addington-Hall J. What Predicts Realisation of Preferred Place of Death in England? Results from a Post-bereavement Survey. 7th World Research Congress of the European Association for Palliative Care (EAPC); 7-9 June 2012; Trondheim, Norway: Palliative Medicine; 2012. p. 440-1.

32. Hunt KJ, Shlomo N, Addington-Hall J. End-of-life care and achieving preferences for place of death in England: Results of a population-based survey using the VOICES-SF questionnaire. Palliat Med. 2014a;28(5):412-21. doi: 10.1177/0269216313512012.

33. Hunt KJ, Shlomo N, Addington-Hall J. End-of-Life Care and Preferences for Place of Death among the Oldest Old: Results of a Population-Based Survey Using VOICES–Short Form. J Palliat Med. 2014b;17(2):176-82. doi: 10.1089/jpm.2013.0385.

34. Ingleton C, Morgan J, Hughes P, Noble B, Evans A, Clark D. Carer satisfaction with end‐of‐life care in Powys, Wales: a cross‐sectional survey. Health & social care in the community. 2004;12(1):43-52.

35. Ipsos MORI. Attitudes towards death and dying in the east of England - Quantitative Report. NHS, East of England: Ipsos MORI. 2010.

36. Johnson S, Sherwen E. Preferred Priorities for Care: West Essex Evaluation. Epping: NHS West Essex, 2010.

37. Johnson M, Nunn A, Hawkes T, Stockdale S, Daley A. Planning for end-of-life care in heart failure: experience of two integrated cardiology-palliative care teams. Br J Cardiol. 2012;19(2):71-5.

38. King G, Mackenzie J, Smith H, Clark D. Dying at home: evaluation of a hospice rapid-response service. Int J Palliat Nurs. 2000;6(6):280-7. PubMed PMID: MEDLINE:11249449.

39. Koekkoek JAF, Dirven L, Reijneveld JC, Sizoo EM, Pasman HR, Postma TJ, et al. End of life care in high-grade glioma patients in three European countries: a comparative study. J Neurooncol. 2014;120(2):303-10. Epub 2014/07/21. doi: 10.1007/s11060-014-1548-5. PubMed PMID: 25038849.

40. Koffman J, Higginson IJ. Dying to be home? Preferred location of death of first-generation black Caribbean and native-born white patients in the United Kingdom. J Palliat Med. 2004;7(5):628-36.

41. Leadbeater C, Garber J. Dying for Change. London: Demos; 2010.

42. McCall K, Rice AM. What influences decisions around the place of care for terminally ill cancer patients? Int J Palliat Nurs. 2005;11(10):541-7.

43. McKeown A, Agar R, Gambles M, Ellershaw JE, Hugel H. Renal failure and specialist palliative care: an assessment of current referral practice. Int J Palliat Nurs. 2008;14(9):454-8.

44. Newton J. Report of Preferred Place of Care Pilot South Essex End of Year One October 2005-2006. South Essex NHS Palliative and Supportive Care Network.

45. Newton J, Clark R, Ahlquist P. Evaluation of the introduction of an advanced care plan into multiple palliative care settings. Int J Palliat Nurs. 2009;15(11):554-61.

46. NHS Public Health North East. A good death. Time to think: 'A Good Death' Consultation Full Research Findings'. A Good Death: Time to Think, NHS Public Health North East, Better Health: Fairer Health, 2010.

47. Norfolk Health Overview and Scrutiny Committee. How we manage death and dying in Norfolk County and Waveney. Norfolk Health Overview and Scrutiny Committee, 2005.

48. Office for National Statistics. National Bereavement Survey (VOICES) 2012. In: Office for National Statistics, editor. <http://www.ons.gov.uk/ons/rel/subnational-health1/national-bereavement-survey--voices-/2012/stb---national-bereavement-survey-2012.html2013>.

49. Oxenham D, Finucane A, Arnold E, Russell P. Delivering preference for place of death in a specialist palliative care setting. BMJ Qual Improv Rep. 2013;2(1):10.1136/bmjquality.u201375.w897. doi: 10.1136/bmjquality.u201375.w897.

50. Peacock O, Riches K, Frisby J, Lund J. ‘‘The gold record’’; highlighting changes in end of life care in colorectal cancer patients. 2nd Biennial Meeting of the Eurasian Colorectal Technologies Association (ECTA) 15-17 June 2011; Turin, Italy. Techniques in Coloproctology: Techniques in Coloproctology; 2011. p. 215-53.

51. Pearse H, Saxby C, Hicks F, Kite S. Patient choice regarding place of death. Palliat Med. 2005;19(2):171-2.

52. Shucksmith J, Carlebach S, Whittaker V. Dying: Discussing and planning for end of life NatCen Social Research, 2013.

53. Sives DA, Cornbleet MA, Murray SA. Preferred place of care - are we asking the question? 7th Palliative Care Congress; 29th April – 1st May 2008; Glasgow, Scotland. Palliative Medicine: Palliative Medicine; 2008. p. 578.

54. Swindlehurst H, Walton W-J, Oldacre M, Evans U. Analysis of the Gold Standards Framework monitoring of community palliative and end of life care. Shrewsbury: Shropshire County Primary Care Trust; Telford and Wrekin Primary Care Trust, 2006.

55. Thomas C, Morris SM, Clark D. Place of death: preferences among cancer patients and their carers. Soc Sci Med. 2004;58(12):2431-44. doi: 10.1016/j.socscimed.2003.09.005. PubMed PMID: WOS:000221190500005.

56. Thompson-Hill J, Hookey C, Salt E, O’Neill T. The supportive care plan: a tool to improve communication in end-of-life care. Int J Palliat Nurs. 2009;15(5):250-5.

57. Waghorn M, Young H, Davies A. Opinions of patients with cancer on the relative importance of place of death in the context of a ‘good death’. BMJ Support Palliat Care. 2011;1(3):310-4. doi: 10.1136/bmjspcare-2011-000041.

58. Walker S, Read S, Priest H. Identifying, documenting, and reviewing preferred place of death: an audit of one UK hospice. Int J Palliat Nurs. 2011;17(11):546-51. PubMed PMID: MEDLINE:22240632.

59. Weir M, Magowan L, Doherty M, Sharkey R, Mc Closkey M, Kelly M, et al. P194 Why do lung cancer patients still die in hospital? British Thoracic Society Winter Meeting 2011; London, UK. Thorax: Thorax; 2011. p. A146.

60. Wood CD, Salter JD. A time and a place: What people want at the end of life. Sue Ryder, 2013.

61. Wood J, Storey L, Clark D. Preferred place of care: an analysis of the 'first 100' patient assessments. Palliat Medicine. 2007;21(5):449-50. Epub 2007/09/29. doi: 10.1177/0269216307078294. PubMed PMID: 17901105.
