# Supplementary material for: Do Patients Want to Die at Home? A Systematic Review of the UK Literature, Focused on Missing Preferences for Place of Death
Source: PLoS One. 2015 Nov 10;10(11):e0142723. doi: 10.1371/journal.pone.0142723 (PMC4640665; doi:10.1371/journal.pone.0142723)
Supplement: S2 Table — (DOC) [file pone.0142723.s005.doc]

| **Section/topic** | **#** | **Checklist item** | **Reported on page #** |
| --- | --- | --- | --- |
| **TITLE** | | |  |
| Title | 1 | Identify the report as a systematic review, meta-analysis, or both. | Page 1 “Do patients want to die at home? A systematic review of the UK literature, focused on missing preferences for place of death” |
| **ABSTRACT** | | |  |
| Structured summary | 2 | Provide a structured summary including, as applicable: background; objectives; data sources; study eligibility criteria, participants, and interventions; study appraisal and synthesis methods; results; limitations; conclusions and implications of key findings; systematic review registration number. | Page 2 “Abstract  Background – End-of-life care policy has a focus on enabling patients to die in their preferred place; this is asserted for most to be home. This review: assesses patient preferences for place of death, including the extent of unreported preferences; examines the importance of patient factors (place of care and health diagnosis) and who reports preference on where patients wish to die.  Methods and findings - Systematic literature review of 7 electronic databases, grey literature, backwards citations from included studies and Palliative Medicine hand search. Included studies published between 2000-2015, reporting original, quantifiable results of adult UK preferences for place of death. Of 10826 articles reviewed, 61 met the inclusion criteria. Summary charts present preferences for place of death by health diagnosis, where patients were asked and who reported the preference. These charts are recalculated to include ‘missing data,’ the views of those whose preferences were not asked, expressed or reported or absent in studies. Missing data were common. Across all health conditions, the majority preference was for home: when missing data were included, home became the minority preference for patients with cancer, non-cancer or multiple conditions. Patients, family proxies and public all expressed a majority preference for home: when missing data were included, home became the minority preference for patients and family proxies. Where patients wished to die was related to where they were asked their preference. Missing data calculations are limited to ‘reported’ data.  Conclusions – It is unknown what proportion of patients prefer to die at home. Reported preferences for place of death often exclude the views of those with no preference or not asked: when ‘missing data’ are included, they supress the proportion of preferences for all locations. It is not therefor valid to assert that the majority of patients prefer to die at home. |
| **INTRODUCTION** | | |  |
| Rationale | 3 | Describe the rationale for the review in the context of what is already known. | Page 3 “Home is stated to be where most patients want to die because, as the foreword of the 2008 EOLC Strategy reports, “From surveys of the general public we know that, given the opportunity and right support, most people would prefer to die at home.”[1] The extent to which the preferences of the general public reflect the views of dying patients is unclear; the two viewpoints may vary due to differences in priorities[4] and whether preferences asked are hypothetical or of practical significance.[5]  It is also unknown how ‘missing’ preferences (the views of participants with no clear preference, or who were unwilling or unable to express or communicate a preference) are treated.[6] Patient preferences for place of death are often ill-defined and evolve as their health deteriorates and their needs change.[7] Preferences for place of death are not categorical choices; they are highly contingent and dependent on the support available.[8] Respondents to questionnaires about preferences are typically though restricted to choices of home, hospice, care home or hospital, whether the questions are asked of the general public[9] or dying patients[10] and it unclear how those whose preferences do not fit these choices are included in study reporting. Excluding ‘missing’ preferences, while not a problem of the magnitude as that of unpublished clinical trials,[11] has the potential to significantly misrepresent patient views and hide the nuances of the PPOD decision-making process.  We therefore undertook a new systematic review of literature concerning the preferences of UK respondents for place of death, with a particular focus on the inclusion of “missing preferences”- participants excluded from analysis since their preference was not asked, expressed or reported.” |
| Objectives | 4 | Provide an explicit statement of questions being addressed with reference to participants, interventions, comparisons, outcomes, and study design (PICOS). | Page 3-4 “We therefore undertook a new systematic review of literature concerning the preferences of UK respondents for place of death, with a particular focus on the inclusion of “missing preferences”- participants excluded from analysis since their preference was not asked, expressed or reported. Higginson and Sen-Gupta’s [12] 2000 international review of PPOD of advanced cancer patients is widely cited in the early EOLC literature and reflects the attention at that time on cancer care. Unlike that review we included all diagnoses to follow the current focus on palliative care for all patients, and restricted the literature to UK populations in order to standardise the context. We explored the variation in preferences by participant’s health condition, who reported the preference and where they were asked their preferences. Since others have reported few high quality UK papers on preferences for place of death since 2000,[5] we applied a well-respected study quality measure and included the ‘grey literature.’[13].” |
| **METHODS** | | |  |
| Protocol and registration | 5 | Indicate if a review protocol exists, if and where it can be accessed (e.g., Web address), and, if available, provide registration information including registration number. | Not registered |
| Eligibility criteria | 6 | Specify study characteristics (e.g., PICOS, length of follow-up) and report characteristics (e.g., years considered, language, publication status) used as criteria for eligibility, giving rationale. | Page 4 “To move beyond the current policy assumption of PPOD we considered preferences in three ways; whether the participant had a malignant diagnosis, whose preference was reported, and where participants were asked their preference. The role of disease is pertinent because UK EOLC provision has historically focused on meeting the needs of cancer patients,[1] and therefore it is plausible that patients with other conditions may have different end of life preferences which are not as well recognised. The role of a participant could have an implication on the answer given when asked about PPOD. As outlined in the Introduction, how preferences are considered may be different for a dying patient compared to a family member acting as a proxy for a dying or deceased patient or a member of the public. The place of an EOLC participant’s care could also be related to their PPOD; patients experiences of care settings have been shown to be a contextualising factor in where they choose to die.[13] Since place of care is not always documented, we used the proxy measure of where participants were asked their preference. |
| Information sources | 7 | Describe all information sources (e.g., databases with dates of coverage, contact with study authors to identify additional studies) in the search and date last searched. | Page 5 “An initial scoping search was carried out (see S1Box for initial search strategy) and the results were reviewed with the below inclusion/exclusion criteria. The search strategy was then revised and improved with the guidance of the review team’s Information Scientist (IK) (see Fig. 1 for search strategy). Searches for papers published between 2000 and January 2015 were carried out in Medline, Embase, PsycINFO (all via OVID), CINAHL (via EbscoHOST), Web of Science, Scopus, ASSIA (via Proquest) and the results were reviewed with the same criteria. A comprehensive search of grey literature identified other studies published over this period; relevant databases and websites of government policy, policy institutes and charities were reviewed.  Fig. 1. Search Strategy  Hand search of Palliative Medicine, the most prevalent journal for included articles, screening of relevant review papers, citation searches of all included papers for other peer reviewed papers, and the authors’ prior knowledge completed the search strategy..”  Box 1 |
| Search | 8 | Present full electronic search strategy for at least one database, including any limits used, such that it could be repeated. | Box 1 |
| Study selection | 9 | State the process for selecting studies (i.e., screening, eligibility, included in systematic review, and, if applicable, included in the meta-analysis). | Page 5 “Inclusion and exclusion criteria  Articles were included in the review if they were: published after 1999, written in English, conducted in a UK setting, and reported quantifiable, empirical data on adults’ preferences for place of death. We restricted the literature to UK populations to increase the homogeneity of health service and cultural context. Likewise, we focused on adult preferences only, recognising the differences in EOLC for children.[1] Opinion pieces, conference abstracts and news reports were excluded unless they contained original empirical data.” |
| Data collection process | 10 | Describe method of data extraction from reports (e.g., piloted forms, independently, in duplicate) and any processes for obtaining and confirming data from investigators. | Page 5-6 “Data were extracted from each included paper and tabulated in Microsoft Excel (by SH or ZM).” |
| Data items | 11 | List and define all variables for which data were sought (e.g., PICOS, funding sources) and any assumptions and simplifications made. | Page 6 “Separate spreadsheets categorised studies by: main diagnosis of participants (cancer, non-cancer, multiple conditions, not stated and ‘public’ to refer to participants surveyed as a member of the general public); who reported the preference (patients, family or other informal carer, healthcare professionals and public) and setting (care home, home, hospice, hospital, multiple settings or ‘not applicable’ as participants were not patients or reporting on behalf of patients).To explore the impact of ‘missing data,’ these spreadsheets were then reproduced with preference percentages including participants whose preferences were either not recorded in the study or not reported in the paper. The ‘missing’ preferences were included as a discrete category since they could not accurately be included in any of the other pre-existing preference categories. Both sets of spreadsheets were plotted as bar charts with lines superimposed indicating median, maximum and minimum home preferences for each category (see Figs. 4-6).” |
| Risk of bias in individual studies | 12 | Describe methods used for assessing risk of bias of individual studies (including specification of whether this was done at the study or outcome level), and how this information is to be used in any data synthesis. | Page 6 “Each included paper was then weighted for its contribution towards answering the review question using Gough’s Weight of Evidence Framework [13]. Details of the Framework and the weighting method are shown in Fig. 3. Each paper was weighted independently by two authors (SH and ZM or SB) with differences in scores reconciled through discussion. A sensitivity analysis of included papers investigated the impact of removing lowest weighted papers, but found no meaningful change in results; we therefore report analysis of all included papers.” |
| Summary measures | 13 | State the principal summary measures (e.g., risk ratio, difference in means). | Page 6 “Both sets of spreadsheets were plotted as bar charts with lines superimposed indicating median, maximum and minimum home preferences for each category (see Figs. 4-6)” |
| Synthesis of results | 14 | Describe the methods of handling data and combining results of studies, if done, including measures of consistency (e.g., I^2^) for each meta-analysis. | Page 6-7 “Data were extracted from each included paper and tabulated in Microsoft Excel (by SH or ZM). Separate spreadsheets categorised studies by: main diagnosis of participants (cancer, non-cancer, multiple conditions, not stated and ‘public’ to refer to participants surveyed as a member of the general public); who reported the preference (patients, family or other informal carer, healthcare professionals and public) and setting (care home, home, hospice, hospital, multiple settings or ‘not applicable’ as participants were not patients or reporting on behalf of patients).To explore the impact of ‘missing data,’ these spreadsheets were then reproduced with preference percentages including participants whose preferences were either not recorded in the study or not reported in the paper. The ‘missing’ preferences were included as a discrete category since they could not accurately be included in any of the other pre-existing preference categories. Both sets of spreadsheets were plotted as bar charts with lines superimposed indicating median, maximum and minimum home preferences for each category (see Figs. 4-6).  Where several different samples were reported in the papers, only that most relevant to the review were included. Where possible this was the ‘total’ preference for place of death of the entire sample; where this was not reported it was calculated where feasible from the available data. Where this was neither possible nor appropriate, for example where data were reported from different years, we report both populations. Preferences for ‘don’t mind’ and ‘no preference’ were merged. Likewise, where participants were not decided or were reported to have a preference of ‘changed mind,’ this was categorised as ‘Unsure/it depends.’ Reported non-responses were categorised as ‘missing’ unless a reason was given which meant that the response could be otherwise categorised (e.g. reported as ‘undecided’). References to nursing or residential home were categorised as ‘care home.’  Broad inclusion criteria ensured that all relevant data could be included and the review studies were diverse in terms of: populations and settings; sampling methods and sample sizes; and research methods. Due to this considerable heterogeneity it was not possible to undertake a meta-analysis to provide an overall percentage for home death preference, nor to test statistically the relationships between categories and preferences for place of death. As the data were not normally distributed, medians rather than means are presented.” |

Page 1 of 2

| **Section/topic** | **#** | **Checklist item** | **Reported on page #** |
| --- | --- | --- | --- |
| Risk of bias across studies | 15 | Specify any assessment of risk of bias that may affect the cumulative evidence (e.g., publication bias, selective reporting within studies). | Page 5 “Each included paper was then weighted for its contribution towards answering the review question using Gough’s Weight of Evidence Framework [13]. Details of the Framework and the weighting method are shown in Fig. 3. Each paper was weighted independently by two authors (SH and ZM or SB) with differences in scores reconciled through discussion. A sensitivity analysis of included papers investigated the impact of removing lowest weighted papers, but found no meaningful change in results; we therefore report analysis of all included papers.” |
| Additional analyses | 16 | Describe methods of additional analyses (e.g., sensitivity or subgroup analyses, meta-regression), if done, indicating which were pre-specified. | Page 6 “A sensitivity analysis of included papers investigated the impact of removing lowest weighted papers, but found no meaningful change in results; we therefore report analysis of all included papers.” |
| **RESULTS** | | |  |
| Study selection | 17 | Give numbers of studies screened, assessed for eligibility, and included in the review, with reasons for exclusions at each stage, ideally with a flow diagram. | Figure 1  Page 7 “The initial search strategy yielded 1,973 titles, the full strategy 8,853 titles and the results were combined. The results of the grey literature search and additional searches were then incorporated at the end of reviewing process. 61 reports met the review inclusion criteria. Three reported duplicate data,[14, 15]; [16, 17]; and [18, 19] and were combined for analysis leaving 58 discrete papers. Several studies reported datasets of multiple populations; three contained two datasets and therefore were included twice[20-22] and two studies generated three reports[23],[24] and were thus represented three times each. This brought the total number of included reports to 65.” |
| Study characteristics | 18 | For each study, present characteristics for which data were extracted (e.g., study size, PICOS, follow-up period) and provide the citations. | Table 2  Page 8-9 “The fifty-eight included studies were research papers (n=34), poster abstracts (n=8), reports (n=9) (including 4 NHS reports), letters to Editors (n=5), a conference abstract (n=1) and a website report (n=1). There was large variation in studies’ aims. Some were concerned with measurement of concordance between preferred and actual place of death, others the evaluation of service redesign on place of death, still others were audits of current preferences to inform service redesign and there were also population studies seeking to inform EOLC policy.  Diverse approaches for data gathering were used. Patient records (n=32) were commonly consulted often from Preferred Priorities for Care (PPC) documents (n=11) (an advance care planning tool designed to encourage the discussion, recording and implementation of patient preferences). Others used questionnaires and surveys (n=19), interviews (n=5) or a combination of these methods (n=2).  Most reports (n=21) included a range of cancer and non-cancer illnesses. Some (n=12) did not state the participants’ illnesses, some studied only cancer (n=8) or specific non-cancer conditions (n=7).[27-31] None of the studies of the general population (n=10) had a disease focus.  Most reports were of patient preferences (referred from here on as ‘participants’) (n=48), of which a minority were proxy reports from family carers (n=11) or healthcare professionals (n=1). Ten were surveys of the general population (referred to from here on as ‘public’).  Data collection was undertaken in varied settings; hospital (n=10), hospices (n=8), participants’ homes (n=5), care homes (n=2) and in the ‘community’ (GP surgeries or a variety of non-acute settings) (n=6). Studies were also undertaken in ‘multiple’ settings where participants were asked in either primary and secondary care or where the participant was responding on behalf of a patient (n=17), or among the general population where location was not relevant (chart category ‘N/A’ (n=10)). ” |
| Risk of bias within studies | 19 | Present data on risk of bias of each study and, if available, any outcome level assessment (see item 12). | Page 6 “Each included paper was then weighted for its contribution towards answering the review question using Gough’s Weight of Evidence Framework [13]. Details of the Framework and the weighting method are shown in Fig. 3. Each paper was weighted independently by two authors (SH and ZM or SB) with differences in scores reconciled through discussion. A sensitivity analysis of included papers investigated the impact of removing lowest weighted papers, but found no meaningful change in results; we therefore report analysis of all included papers.”  Table 2 |
| Results of individual studies | 20 | For all outcomes considered (benefits or harms), present, for each study: (a) simple summary data for each intervention group (b) effect estimates and confidence intervals, ideally with a forest plot. | Table 2  Figs. 4-6 |
| Synthesis of results | 21 | Present results of each meta-analysis done, including confidence intervals and measures of consistency. | Not applicable |
| Risk of bias across studies | 22 | Present results of any assessment of risk of bias across studies (see Item 15). | Page 6 “Each included paper was then weighted for its contribution towards answering the review question using Gough’s Weight of Evidence Framework [13]. Details of the Framework and the weighting method are shown in Fig. 3. Each paper was weighted independently by two authors (SH and ZM or SB) with differences in scores reconciled through discussion. A sensitivity analysis of included papers investigated the impact of removing lowest weighted papers, but found no meaningful change in results; we therefore report analysis of all included papers..”  Table 2  Figs. 4-6 |
| Additional analysis | 23 | Give results of additional analyses, if done (e.g., sensitivity or subgroup analyses, meta-regression [see Item 16]). | Page 6 “Each included paper was then weighted for its contribution towards answering the review question using Gough’s Weight of Evidence Framework [13]. Details of the Framework and the weighting method are shown in Fig. 3. Each paper was weighted independently by two authors (SH and ZM or SB) with differences in scores reconciled through discussion. A sensitivity analysis of included papers investigated the impact of removing lowest weighted papers, but found no meaningful change in results; we therefore report analysis of all included papers.” |
| **DISCUSSION** | | |  |
| Summary of evidence | 24 | Summarize the main findings including the strength of evidence for each main outcome; consider their relevance to key groups (e.g., healthcare providers, users, and policy makers). | Page 8-9 “The fifty-eight included studies were research papers (n=34), poster abstracts (n=8), reports (n=9) (including 4 NHS reports), letters to Editors (n=5), a conference abstract (n=1) and a website report (n=1). There was large variation in studies’ aims. Some were concerned with measurement of concordance between preferred and actual place of death, others the evaluation of service redesign on place of death, still others were audits of current preferences to inform service redesign and there were also population studies seeking to inform EOLC policy.  Diverse approaches for data gathering were used. Patient records (n=32) were commonly consulted often from Preferred Priorities for Care (PPC) documents (n=11) (an advance care planning tool designed to encourage the discussion, recording and implementation of patient preferences). Others used questionnaires and surveys (n=19), interviews (n=5) or a combination of these methods (n=2).  Most reports (n=21) included a range of cancer and non-cancer illnesses. Some (n=12) did not state the participants’ illnesses, some studied only cancer (n=8) or specific non-cancer conditions (n=7).[27-31] None of the studies of the general population (n=10) had a disease focus.  Most reports were of patient preferences (referred from here on as ‘participants’) (n=48), of which a minority were proxy reports from family carers (n=11) or healthcare professionals (n=1). Ten were surveys of the general population (referred to from here on as ‘public’).  Data collection was undertaken in varied settings; hospital (n=10), hospices (n=8), participants’ homes (n=5), care homes (n=2) and in the ‘community’ (GP surgeries or a variety of non-acute settings) (n=6). Studies were also undertaken in ‘multiple’ settings where participants were asked in either primary and secondary care or where the participant was responding on behalf of a patient (n=17), or among the general population where location was not relevant (chart category ‘N/A’ (n=10)). ” |
| Limitations | 25 | Discuss limitations at study and outcome level (e.g., risk of bias), and at review-level (e.g., incomplete retrieval of identified research, reporting bias). | Page 14-5 “Our calculations for missing data are limited to ‘reported’ missing data, which is where population and sample sizes are detailed. Public opinion surveys were particularly likely to not report the number of participants who did not state a preference for place of death. In these studies it was not possible to present recalculated preferences to reflect ‘missing’ data, and this may explain some of the difference between the homogeneity of public preferences against the heterogeneity of patient preferences. Likewise some audit studies of PPC documents did not report how many records they viewed which did not have information on PPOD (e.g.[42]). Where studies included only a set number of responses (e.g.[43]), we do not know how many participants were not asked their preferences. For some papers, calculations of preferences including ‘missing’ data are only estimates as only weighted preferences for place of death were reported.  The distribution of “missing” preferences is unknown and it is therefore not possible to speculate on where these participants would have preferred to die. These unknown preferences could represent participants not having a preference at all. Missing preferences may also represent having a PPOD other than home, or they could support the policy rhetoric that most dying patients wish to be at home.  When the missing data was included, the proportions of preferences for all locations were reduced. Consequently, of the known preferences, the majority of respondents still preferred home over other locations as a place of death, thereby supporting the current policy focus. However, this interpretation overlooks the scale of the missing data.  The heterogeneity of our sample of patient and carer studies may explain the variations of the responses identified, rather than being an inherent feature of these preferences for place of death.[5] The association between where participants were asked about preferences and their PPOD does not imply causality; participants’ location may have already been their PPOD, rather than the experience of where they were asked affecting their PPOD.” |
| Conclusions | 26 | Provide a general interpretation of the results in the context of other evidence, and implications for future research. | Page 10  “In many reports there was a large proportion of missing participants’ preferences. The ‘missing’ preferences are likely to represent preferences that were not asked or not expressed. Preferences may have been missing because participants may not have been given the opportunity to state their preference, and so could reflect the difficulty healthcare professionals have in holding EOLC conversations.[34] Such ‘missing’ preferences could therefore denote unrecorded preferences for death in any setting. Preferences may also be missing because participants did not have a preference to give. Missing preferences like these suggest that participants prioritised other EOLC issues such as pain and symptom management over place of death.[4]Participants may also have been excluded from analysis because they; were undecided about where they wished to die, did not wish to talk about preferences for place of death, were impaired cognitively or physically from communicating their preference or did not have their preference recorded.[35] Regardless of the reasons, the exclusion of ‘missing’ preferences from study reporting inflates the significance of recorded preferences.  We could only demonstrate that a minority of participants with cancer preferred home as their place of death, due the extent of missing data. EOLC provision has historically focused on the needs of cancer patients,[1] and so it is of consequence that even this ‘privileged’ group’s preferences cannot be said to be known to correlate with the policy rhetoric of home as the majority PPOD.  The variance we found between the preferences of the general public and patients is reported by others,[5] and could in part be explained by differences in data collection. For example, information provided about the general public was often drawn from large surveys whilst patient preferences were often collected from patient records. The dissimilarity between public and patient preferences may also in part be attributed to the different meanings given by respondents to questions about PPOD. It is plausible that members of the public asked a hypothetical question[36] concerning a terminal illness may give a different response to a patient who is actually dying. Patient preferences for place of death have been shown to be highly contingent and part of a process rather than absolute a priori decisions.[8]  Family members’ proxy reports of patients’ PPOD contained a large amount of missing data. One reason for this may partially be due to compromised recall because of the period between bereavement and when participants are asked to take part in research.[37] It may also in part be due to a reluctance of patients to disclose their PPOD to relatives; either because they did not wish to be a burden,[38] or because where they died was not a high priority for them.[4]  Where participants were asked about their PPOD appeared broadly associated with where they wished to die. We cannot ascertain causation from this association; it may be because participants were in their preferred place of care when asked their preference. The association could also suggest that preferences are contextualised by patients’ experience of care[35] rather than being an isolated choice and therefore may demonstrate that patients prefer familiarity over change at the end of life.[39]  The review has demonstrated a substantial amount of missing data on UK participants’ preferences for place of death. We do not know what locations, if any, these ‘missing’ preferences are for and it is therefore not valid to assert that the majority of patients wish to die at home.  The extent of missing data has major implications for clinical practice. Some patients may have preferences that are not elicited, calling for sensitive communication skills to encourage them to express their views. Others may not wish to express their preference, or may have no preference and this should be respected.[40] Healthcare professionals, researchers, policy makers and others involved in the care of dying patients need to recognise that not having a preference for place of death is a legitimate opinion which should be recorded in the same way as preferences for specific locations.[6] Including a “missing” preference as a valid category for reporting and analysis would also aid future research.  Whilst general public surveys are valuable in assessing public opinion, they do not appear to reflect dying patient preferences. Likewise, family members appear to be poor proxies for patient PPOD. This has implications for UK health policy which relies heavily on next-of-kin reports, particularly the national survey of bereaved people (“VOICES: Views of Informal Carers – Evaluation Of Services” [41]). The association between where participants were asked their preferences and their PPOD, regardless of the direction of inference, suggests that caution is needed in assuming that home should be the default location for future care for dying patients who are currently being cared for in other settings.” |
| **FUNDING** | | |  |
| Funding | 27 | Describe sources of funding for the systematic review and other support (e.g., supply of data); role of funders for the systematic review. | Our funding statement is provided in the online submission form and reads “This article presents independent research funded by the National Institute for Health Research (NIHR) Collaboration for Leadership in Applied Health Research & Care (CLAHRC) East of England and previously CLAHRC Cambridge and Peterborough, grant number RG74481 http://www.clahrc-eoe.nihr.ac.uk/. The views expressed are those of the authors and not necessarily those of the NHS, the NIHR or the Department of Health. The NIHR provided support in the form of salaries for authors (SH, ZM and SB) but had no role in study design, data collection and analysis, decision to publish, or preparation of the manuscript.” |

*From:*  Moher D, Liberati A, Tetzlaff J, Altman DG, The PRISMA Group (2009). Preferred Reporting Items for Systematic Reviews and Meta-Analyses: The PRISMA Statement. PLoS Med 6(6): e1000097. doi:10.1371/journal.pmed1000097

For more information, visit: **www.prisma-statement.org**.
